# Functional analysis of conserved *C*. *elegans* bHLH family members uncovers lifespan control by a peptidergic hub neuron

**DOI:** 10.1371/journal.pbio.3002979

**Published:** 2025-01-06

**Authors:** G. Robert Aguilar, Berta Vidal, Hongzhu Ji, Joke Evenblij, Chien-Po Liao, Hongfei Ji, Giulio Valperga, Christopher Fang-Yen, Oliver Hobert

**Affiliations:** 1 Department of Biological Sciences, Howard Hughes Medical Institute, Columbia University, New York, New York, United States of America; 2 Technische Universität, Braunschweig, Germany; 3 Department of Biomedical Engineering, Ohio State University, Columbus, Ohio, United States of America; University of Notre Dame, Center for Stem Cells and Regenerative Medicine, UNITED STATES OF AMERICA

## Abstract

Throughout the animal kingdom, several members of the basic helix-loop-helix (bHLH) family act as proneural genes during early steps of nervous system development. Roles of bHLH genes in specifying terminal differentiation of postmitotic neurons have been less extensively studied. We analyze here the function of 5 *Caenorhabditis elegans* bHLH genes, falling into 3 phylogenetically conserved subfamilies, which are continuously expressed in a very small number of postmitotic neurons in the central nervous system. We show (a) that 2 orthologs of the vertebrate *bHLHe22/e23* genes, called *hlh-17* and *hlh-32*, function redundantly to specify the identity of a single head interneuron class (AUA), as well as an individual motor neuron (VB2); (b) that the *PTF1a* ortholog *hlh-13* acts as a terminal selector to control terminal differentiation and function of the sole octopaminergic neuron class in *C*. *elegans*, RIC; and (c) that the *NHLH1/2* ortholog *hlh-15* controls terminal differentiation and function of the peptidergic AVK head interneuron class, a known neuropeptidergic signaling hub in the animal. Strikingly, through null mutant analysis and cell-specific rescue experiments, we find that loss of *hlh-15/NHLH* in the peptidergic AVK neurons and the resulting abrogation of neuropeptide secretion from these neurons causes a substantially extended lifespan of the animal, which we propose to be akin to hypothalamic control of lifespan in vertebrates. Our functional analysis reveals themes of bHLH gene function during terminal differentiation that are complementary to the earlier lineage specification roles of other bHLH family members. However, such late functions are much more sparsely employed by members of the bHLH transcription factor family, compared to the function of the much more broadly employed homeodomain transcription factor family.

## Abstract 2

Members of the conserved bHLH gene family have diverse roles during the development of the nervous system, but how they specify neuronal terminal differentiation remains unclear. Here, the authors analyze the function of five C. elegans bHLH genes, showing that some can regulate neuropeptide secretion and significantly expand lifespan when mutated.

## Introduction

Basic helix-loop-helix (bHLH) transcription factors constitute a large, deeply conserved family of transcription factors with wide-ranging roles in animal development and physiology (reviewed in [1–7]). bHLH proteins have been categorized into 6 higher order groups (Group A to F) based on intrinsic sequence features of the bHLH domain and/or the presence of additional domains [8–10]. Group A is the largest of these groups and contains proteins with deeply conserved developmental patterning functions in several tissue types, most prominently in the nervous system (e.g., Atonal, ASC families) and muscle (MyoD, Twist). Many members of this group heterodimerize with a common subunit, the E-proteins in vertebrates, Daughterless in *Drosophila* and HLH-2 in *Caenorhabditis elegans*, which are also all members of group A. This common subunit is often referred to as a “class I” bHLH protein, while other group A members that heterodimerize with the class I protein are referred to as “class II” bHLH proteins [7]. While members of all major bHLH groups (i.e., group A to F) appear to have been present in single-celled organisms [11], group A bHLH genes underwent major expansions during metazoan evolution [10] and hence are possible contributors to animal cell type diversification.

**Fig 1:**
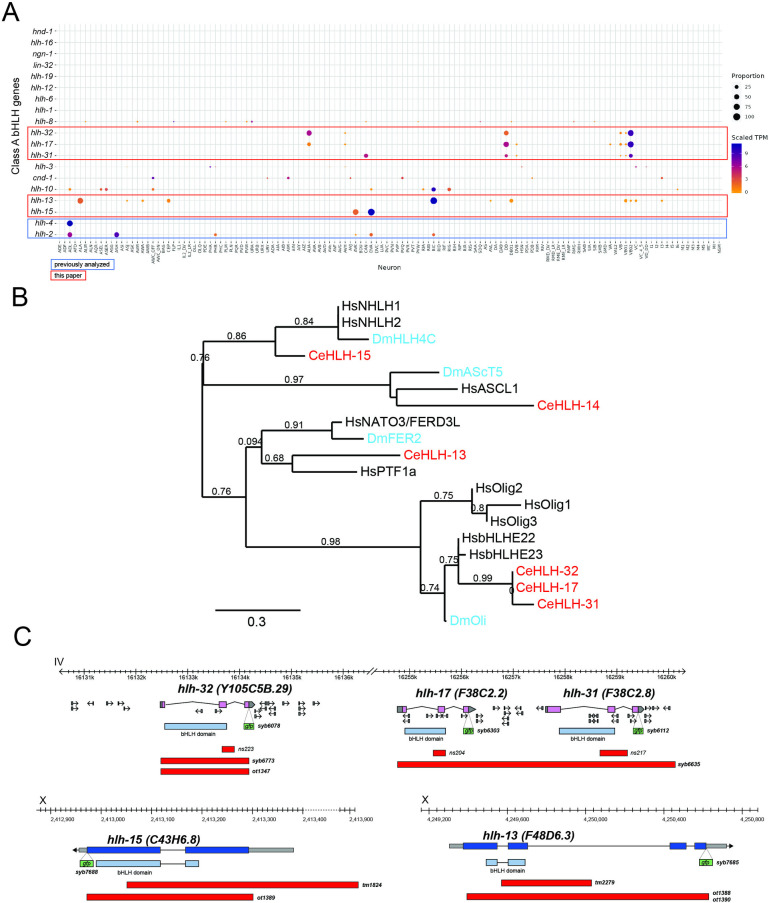
Group A class members in *C*. *elegans*. (**A**) Expression of group A class members in *C*. *elegans* in the CeNGEN scRNA-seq data set [19]. Orthologs are as follows [8,9]: *hlh-2 = E12/47*, D.m. Daughterless; *hlh-1 = MyoD*; *hlh-3*, *4*, *6*, *12*, *19* = ASC family; *lin-32 = Atonal/ATO*; *cnd-1 =* NeuroD; *ngn-1 =* Neurogenin; *hlh-17*, *hlh-31*, *hlh-32 =* bHLHe22/e23 (see **S1 Fig**); *hlh-16 =* Olig related (see **S1 Fig**); *hlh-10*, *hlh-12 = TCF21*; *hlh-13 = PTF1a; hlh-15 = NHLH1 and 2*; *hlh-8 =* Twist; *hnd-1 = Hand*. (**B**) Phylogenetic relationship of the bHLH domain sequences of select group A bHLH family members. A representative ASc family member (HLH-14) and its orthologs are included. There are 2 additional FER paralogs in *Drosophila* not included in this tree. Phylogenetic trees were generated at phylogeny.fr [123] with default parameters. (**C**) Locus with deletion and reporter alleles for all examined genes (*hlh-13*, *15*, *17*, *31*, *32*).

The nematode *C*. *elegans* contains 19 group A family members, including orthologs of prominent developmental patterning genes, such as mesodermal patterning genes MyoD (*hlh-1* in *C*. *elegans*) and Twist (*hlh-8*) as well as orthologs of neuronal patterning genes Atonal (*lin-32*), Neurogenin (*ngn-1*), NeuroD (*cnd-1*) and others [8,9] (**Fig 1A**). As exemplified by our comprehensive analysis of homeobox gene expression and function [12–14], we reason that broad, family-wide, and animal-wide analyses of transcription factor families may reveal the presence (or absence) of common themes in their expression and function. Hence, over the past few years, our laboratory has engaged in a systematic analysis of several of the group A bHLH proteins throughout the entire *C*. *elegans* nervous system, revealing common and divergent themes in the function of class II proteins, including the ASC homologs *hlh-14* and *hlh-4*, the Atonal homolog *lin-32*, and the class I Daughterless homolog *hlh-2* [15–18].

In this paper, we have used the recently released single-cell RNA sequencing (scRNA-seq) atlas of the entire, mature nervous system of *C*. *elegans* [19] to choose for functional analysis a set of 5 deeply conserved group A family members. These 5 bHLH genes were chosen based on their very restricted, strong expression in individual, mature cell types of the nervous system, where their expression is maintained throughout the life of the animal (**Fig 1A**) [19]. These genes include the sole *C*. *elegans* ortholog of the vertebrate *PTF1a* and *NATO3* genes, *hlh-13*, the sole *C*. *elegans* ortholog of the vertebrate *NHLH1* and *NHLH2* genes, *hlh-15*, and the 3 *C*. *elegans* orthologs of the vertebrate *bHLHe22/e23* genes, called *hlh-17*, *hlh-31*, and *hlh-32* (**Fig 1B**). Each of these vertebrate genes had been implicated in neuronal differentiation in select parts of the mammalian nervous system [20–29]. We make use of the more compact nature of the *C*. *elegans* nervous system to garner a systems-wide view of the functions of their *C*. *elegans* orthologs using the many tools that *C*. *elegans* offers to dissect neuronal differentiation programs. Our analysis is a further stepping-stone in our attempt to generate an encyclopedic map of neuronal identity regulators in *C*. *elegans*. One emerging view of such encyclopedic analyses is that, compared to another prominent transcription factor family, the homeodomain family, bHLH protein function appears more sparsely employed during late differentiation steps in the nervous system.

## Material and methods

### *C*. *elegans* strains

All strains used in this study are listed in **S1 Table**. Strains were maintained as previously described [30].

### *C*. *elegans* genome-engineered strains

Deletion alleles for *hlh-32(ot1347)*, *hlh-13(ot1388)*, *hlh-13(ot1390)*, and *hlh-15(ot1389)* were generated using CRISPR/Cas9 genome engineering [31]. Multiple alleles encompassing the same genomic regions, as shown in **Fig 1C**, represent the same deletion generated in different backgrounds. Deletions were made using 2 crRNAs repaired with an ssODN, as described below:

*hlh-32(ot1347)*: crRNAs (tttcagggtgtcgttagatt and catccgaaggagattagcgc), ssODN (cgctggtgatagattcctggatgttcaacaaattgcgctggtgatagattcctggatgttcaacaaattg)

*hlh-13(ot1388)* and *hlh-13(ot1390)*: crRNAs (gaagctgtcatttacataag and acagagtttgtttaggcaat), ssODN (accaattacaattgtgaattcgagcagaaccacttGCCTAAacaaactctgtgtatgcgtaaacggcaga)

*hlh-15(ot1389)*: crRNAs(ttgaatagtggaacacttgc and tgaattcacaaccttcacag), ssODN(atctgttactcgttttcctatcctctattccagcacagtggccaaccgatatatatatatatatagttcc)

A number of CRISPR/Cas9-engineered reporter and deletion strains were generated by SunyBiotech (indicated by the *syb* allele designation; see **S1 Table**). These include C-terminal *gfp* reporter insertions for all the 5 bHLH genes examined here and SL2::gfp::H2B reporter cassette insertions at the C-terminus of genes that serve as cell fate markers and/or potential targets of bHLH genes (*nlp-49*, *kcc-3*, *col-105*, *pdf-1*, *flp-7*, *bcat-1*). All other previously published cell fate markers used here are also listed in **S1 Table**.

### *C*. *elegans* transgenic strains

To establish the *sshk-1p*::*gfp* reporter transgene, a marker for AUA neurons, the 370 bp intergenic region upstream of *sshk-1* was fused to *gfp* followed by the 3′ untranslated region of *unc-54*, as previously described [32]. Primers were: *sshk-1* promoter (370 bp): **fwd** *CTGTTGACTAATCTCACAGC* **rev** *AGGAAAACTTTCAAATGAGAGG*. The amplicon was injected together with a *pha-1* wild-type copy into in a *pha-1(e2123)* mutant strain to generate *otEx8199*.

To generate *flp-1p*::*genomic hlh-15*::*SL2*::*TagRFP*, a 0.4 kb *flp-1* promoter sequence from pCS169 (*flp-1p(trc)*::*ICE*) [33] attached to the full *hlh-15* genomic sequence (*flp-1p*::*hlh-15 genomic fragment* containing exons and introns) was fully synthesized by Azenta Life Sciences and amplified using the following primers: **fwd** *GGAAATGAAATCAGGAAACAGCTATGACCATGAGCTTAATTCCTAAAAACCC* **rev** *GGTGAAAGTAGGATGAGACAGCCGGCCGTTATTGAAGCAAGTTGTCTAGAAAAC*. This amplicon was then ligated to the SL2::TagRFP backbone amplified from pCPL11 (*srab-20p*::*lin-29a*::*SL2*::*TagRFP*) using Gibson cloning. *hlh-15(ot1389)* mutant animals were then injected with the following concentrations of constructs: *flp-1p*::*ghlh-15*::*SL2*::*tagRFP 25 ng/μl*, *Pttx-3*::*GFP 25 ng/μl*, *pBS 150 ng/μl* to generate *otEx8247* and *otEx8248*.

All other previously described transgenic strains used in this study are listed in **S1 Table.**

### Neuronal identification using NeuroPAL

We crossed our bHLH reporter alleles to the NeuroPAL landmark strain *(otIs669)* to pinpoint which neuron types they are expressed [34]. Neuron types were identified based on the NeuroPAL color code they express and the position of their nuclei relative to other neurons. Detailed guides to neuronal identification using NeuroPAL can be accessed through https://www.hobertlab.org/neuropal/.

### Automated worm tracking

Worm tracking was performed at room temperature using a WormTracker 2.0 imaging system [35]. Immediately before the experiment, NGM plates were seeded with a thin lawn (10 μl) of OP50, on which 5 young adult animals at a time were transferred and recorded for 5 min. Tracking videos were analyzed using the WormLab software (MBF Bioscience).

### Defecation assay

Defecation assays were performed as described [36]. Well-fed, young adult worms were singled to NGM plates with a thin lawn of OP50. Before starting the assay, worms were left to acclimate for 10 min. Plates were mounted on a Nikon Eclipse E400 microscope and worms were observed using the 20X DIC objective. A posterior body muscle contraction (pBOC) of the worm signaled the start of the observation period. For 10 min, the pBOC and enteric muscle contraction (EMC) events were counted, including the first pBOC at the start of the assay.

### Staining for lipids

Staining for lipids using Nile Red (NR) was performed following [37]. L4 worms raised at 20°C were harvested by washing with 1× PBST. Worms were pelleted by centrifugation at 560 × *g* for 1 min and supernatant discarded. To the pellet, 100 μl of 40% isopropanol was added and left to incubate for 3 min at room temperature. Worms were again centrifuged at 560 × *g* for 1 min and supernatant was carefully removed.

To prepare the NR stain, 6 μl of 5 mg/ml NR in 100% acetone was freshly added to 1 ml of 40% acetone. Working in the dark, 600 μl of NR stain was added and thoroughly mixed to each worm pellet by inverting the tubes. Samples were rotated in the dark for 2 h at room temperature. Worms were then pelleted at 560 × *g* for 1 min and the supernatant was removed. To remove excess NR stain, samples were incubated with 600 μl of PBST for 30 min in the dark. Samples were centrifuged at 560 × *g* for 1 min and all but 50 μl of supernatant was discarded. Worms were resuspended in the residual supernatant and imaged immediately after staining. Intensity of the lipid stain was measured in Fiji [38].

### Microscopy

Worms were immobilized using 100 mM sodium azide (NaN_3_) and mounted on 5% agarose pads on glass slides. Unless otherwise indicated, worms were imaged at the L4 or young adult stage. Images were acquired using either Zeiss LSM 980 confocal microscope or Axio Imager Z2 compound microscope at 40× magnification unless otherwise specified. Processing of images was done using the Zen (Zeiss) or Fiji [38] software. Fluorescence intensities of reporters were quantified using Fiji.

### Microfluidic locomotory analysis

The time-lapse images (1 frame/second, 5 h recording) for behavioral assays were acquired using a high-resolution, multi-well imaging system [39]. We added 60 μl of liquid NGM buffer (same components as NGM but without agar or peptone) to each well of a 96-well plate (Corning, Inc.), then picked one Day 1 adult animal to each well. Where indicated, the NGM was supplemented with food *E*. *coli* OP50, and the bacteria were added to NGM to a final OD_600_ ≈ 1. Multiple genotypes or conditions being compared were assayed on the same plate.

Image data processing and analysis were performed as in a previous study [40]. Consecutive images were subtracted to generate difference images. We normalized the difference image with the average pixel intensity of the 2 subtracted images. To reduce noise, we applied a Gaussian filter with SD equal to one pixel to the difference image. We then applied a binary threshold of 0.6 to the filtered difference image to determine whether movement occurred at each pixel. We summed up all pixels of the binarized difference image and used the resulting value to define the activity. To calculate the dwelling fraction of worms, we time-averaged the activity data with a smoothing kernel of 5 s and generated a histogram of the time-averaged activity for each worm. We then employed a nonlinear least-squares fitting algorithm to fit each individual worm’s activity histogram to 2 exponential terms (2 free parameters each) and a Gaussian term (3 free parameters). The zero bin on the activity histogram (corresponding to quiescence) was excluded from the histogram before the fitting process. The dwelling fraction of each worm was calculated as the area under the 2 exponential curve components of the fit.

### Compensatory curvature response (CCR) assay

We employed a straight channel microfluidic device to perform the compensatory curvature response (CCR) behavioral assay as described in a previous study [41] (**Fig 9A**). The microfluidic device consists of 2,000 μm-wide open areas and 2 parallel straight channels (60 μm width, 200 μm length, 80 μm height). During the assay, we transferred day 1 adults to a food-free NGM buffer for approximately 5 min to remove bacteria, and then pipetted the animals from the washing buffer into a microfluidic chamber filled with NGM buffer containing 0.1% (by mass) bovine serum albumin (for preventing worms from adhering to chamber surfaces). We recorded behavioral videos (30 frames/second) of each animal in the chamber for about 3 min performing free locomotion in open areas or constrained locomotion with their mid-body in the narrow channels.

The behavioral data from microfluidic experiments were analyzed using methods described previously [41]. For each worm, the normalized bending curvature *K* is calculated as the product of body curvature *k* and the worm body length *L*. To quantify the effect of straight-channel constraint on worm bending curvature amplitude, the whole-body curvature amplitude during constrained locomotion was computed and compared with that of free locomotion. Specifically, we analyzed worm bending curvature dynamics during free locomotion to generate an averaged curvature amplitude profile, *A_free_(s)*, as a function of body coordinate *s* defined such that s = 0 at the head and s = 1 at the tail. We divided video sequences of constrained movement into individual short sequences using a 3 s time window to analyze constrained locomotion. We manually marked the channel position in each image sequence to record the relative position of the constraint to the worm body. The normalized curvature change in response to mid-body constraint was calculated by considering only periods during which the anterior and posterior limits of the narrow channel were consistently within a body coordinate interval between 0.35 and 0.65. The resulting curvature dynamics were denoted as *K_const_*, and the maximum value of |*K_const_(s,t)*| in the time dimension was defined as the curvature amplitude profile of individual periods, denoted as $A_{const}(s)=\underset{t}{\max}\left|K_{const}(s,t)\right|$. The normalized curvature change of each period was represented by $A_{const}(s)/A_{free}(s)-1$. The normalized anterior curvature changes of individual periods was defined as $\langle A_{const}(s)/A_{free}(s)\rangle {|}_{0.1}^{0.3}-1,$ where ($\langle X(s)\rangle {|}_{a}^{b}$ denotes the average of *X* over the interval [a, b]).

### Lifespan and healthspan assays

*Lifespan assays*. Lifespan assays were performed as previously described [42]. One hundred (100) unhatched embryos were put on a plate, with 3 plates for each genotype. Throughout the experiment, plates were kept in dark boxes at 20°C as continuous exposure to light has been shown to decrease worm lifespan [43]. Embryos were left to develop for 3 days until Day 1 of adulthood, after which 15 young adults were gently transferred to each plate, with 7 plates for each genotype. The number of worms that were alive, dead, and censored were recorded every alternate day of adulthood. Censored worms are worms that die due to bagging, desiccation, contamination, or human error. Worms that are immobile and fail to respond to touch with a platinum pick are considered dead. Dead and censored worms were burned once they were recorded. For the first 6 days of adulthood, worms were transferred to a new plate every day to avoid contamination with their progeny. Once worms stopped producing progeny, usually after day 6 of adulthood, they were transferred to new plates every other day. Lifespan was recorded until death of the last worm, which usually occurred between 30 and 45 days of adulthood. In cases of contamination with mold or unwanted bacteria during the assay, all live worms were transferred to clean plates. Statistical analysis, Kaplan–Meier survival analysis, and Mantel–Cox log-rank test were performed using OASIS 2 software (https://sbi.postech.ac.kr/oasis2) [44].

*Pharyngeal pumping rate*. The assay NGM plate was seeded with 50 μl of OP50 1 day prior to the experiment. Ten minutes before the assay commenced, animals were transferred onto the assay plate. Pharyngeal pumping, a marker of health span [45]. was observed using a Nikon SMZ645 dissecting microscope. The pumping rate was calculated by counting the number of pumps over a 20-s interval, then multiplying this value by 3 to obtain the rate per minute.

*Nucleolar size*. To measure hypodermal cell nucleolar size, a prospective marker of lifespan [46], differential interference contrast (DIC) images of hypodermal cells of day 1 animals were acquired at 63× magnification. Nucleolar area was measured using Fiji, following.

## Results

### Group A bHLH family genes in the nervous system of *C*. *elegans*

The *C*. *elegans* genome encodes members of all 6 groups of bHLH genes (Groups A to F) [8–10,47,48]. We focus here on members of group A since its striking expansion in metazoans [10] provides a clear indication of their role in cell type diversification. There are 19 group A genes in *C*. *elegans*, including paralogues of the phylogenetically deeply conserved Ato, ASC, NeuroD, Neurogenin, and MyoD proteins (**Fig 1A**). While several of these group A genes have been previously analyzed for their function within and outside the nervous system [16,17,49–54], others have remained uncharacterized.

Examination of group A bHLH transcript presence in the whole nervous system single-cell RNA atlas of *C*. *elegans*, established by the CeNGEN consortium [19], reveals that only a subset of group A proteins are expressed in the mature *C*. *elegans* nervous system, and each in a very small number of different mature neuron types (**Fig 1A**). These include several genes whose function in nervous system differentiation have not previously been analyzed. We chose the 5 most strongly expressed and previously little-studied genes from this category (**Fig 1B**), the *PTF1a/NATO3* ortholog *hlh-13*, the *NHLH1/2* ortholog *hlh-15*, and the 3 *bHLHe22/e23* orthologs *hlh-17*, *hlh-31*, and *hlh-32*, for an in-depth analysis of their expression and function. First, we used CRISPR/Cas9 genome engineering to tag each of these 5 genes with *gfp* to analyze the expression of the protein product of these loci throughout the whole animal during all life stages, thereby significantly expanding previous transcript and reporter transgene analysis. Second, we used existing or generated new deletion alleles, again using CRISPR/Cas9 genome engineering, to rigorously probe their function. Reporter and mutant alleles for each examined locus are schematically shown in **Fig 1C**.

### The bHLHe22 and bHLHe23 orthologs HLH-17 and HLH-32 proteins are expressed in neurons, but not glia

The *C*. *elegans* genome codes for 3 members of the Olig superfamily of transcription factors, *hlh-17*, *hlh-31*, and *hlh-32*. In vertebrates, this family is defined by 2 closely related subtypes of genes, the highly interrelated, paralogous *Olig1*, *Olig2*, and *Olig3* genes and the 2 highly related *bHLHb4* (now called *bHLHe23*) and *bHLHb5* (now called *bHLHe22*) gene paralogs. While the 3 *C*. *elegans* genes *hlh-17*, *hlh-31*, and *hlh-32* have previously been considered as orthologs of the Olig subbranch of bHLH genes [55], detailed sequence analysis, as well as orthology assignment tools demonstrate that *hlh-17*, *hlh-31*, and *hlh-32* are more closely related to the bHLHe22/e23 subbranch than to the Olig subbranch [56–58] (**Figs 1B and S1A–S1C**). Conversely, another *C*. *elegans* bHLH gene, *hlh-16*, appears to be more closely related to the Olig genes than *hlh-17*, *hlh-31*, and *hlh-32* (**S1A–S1C Fig**).

All 3 bHLHe22/e23 paralogs cluster within a small interval (<150 kb) on chromosome IV and *hlh-17* and *hlh-31* are direct neighbors (**S1D Fig**). The *hlh-17* and *hlh-32* genes display identical bHLH domain-encoding sequences, indicating that they have the same protein dimerization and DNA-binding properties (**S1B Fig**). All 3 genes are the result of species-specific gene duplications within the nematode phylum. Reciprocal BLAST searches show that *C*. *remanei* has at least 6 members of the bHLHe22/e23 subfamily while *C*. *briggsae* has only a single representative (*Cbr-hlh-17*). Nematodes from other clades also do not harbor *hlh-17/31/32* duplications, leading us to conclude that an ancestral single bHLHb4/5 gene has undergone recent duplications within select members of the *Caenorhabditis* genus and then subsequently duplicated once in the vertebrate lineage, to generate *bHLHe22* and *bHLHe23*. The related Olig-like gene *hlh-16* has not undergone gene duplications in nematode genomes, but it has been lost in *Drosophila*, which contains instead a single bHLHe22/e23 representative, somewhat misleadingly called *Oli* [59] (**Figs 1B and S1**).

**Fig 2:**
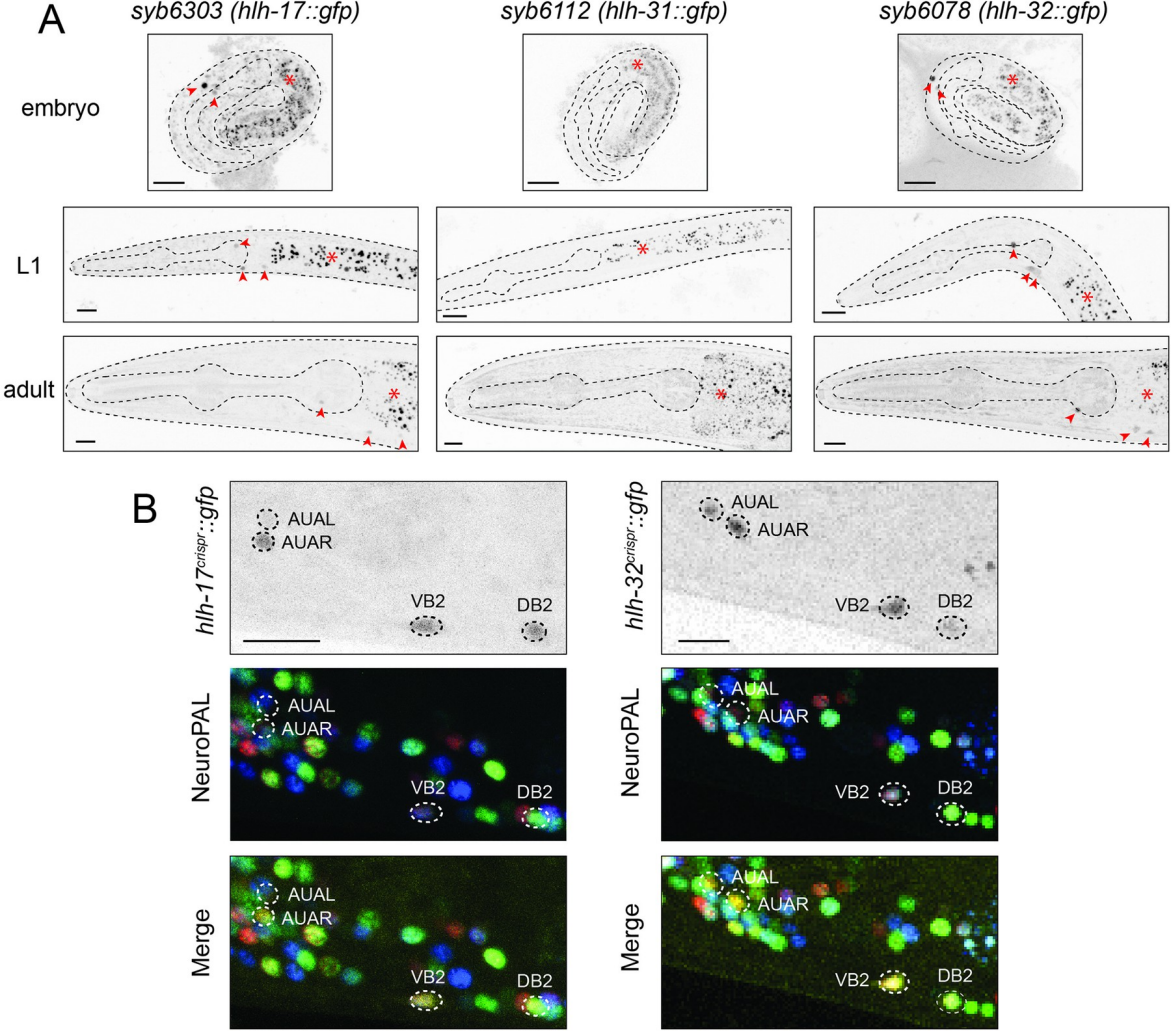
Expression of *hlh-17*, *hlh-31*, and *hlh-32* reporter alleles. (**A**) Expression of CRISPR/Cas9-engineered reporter alleles, *hlh-17(syb6303)*, *hlh-31(syb6112)*, and *hlh-32(syb6078)* over the course of development (see **Fig 1C** for reporter allele). HLH-17::GFP and HLH-32::GFP can be detected in head neurons (red arrowheads), while no HLH-31::GFP signal could be detected anywhere at any stage. Asterisks indicate intestinal autofluorescence. Representative images for reporter alleles are shown with 10 μm scale bar. (**B**) Overlay with the NeuroPAL *(otIs669)* transgene demonstrates *hlh-17(syb6303)* and *hlh-32(syb6078)* expression in the AUA, VB2, and DB2 neurons. Representative close-up views for reporter alleles are shown with 10 μm scale bar.

The fusion of 5′ promoter sequences of the *hlh-17*, *hlh-31*, and *hlh-32* genes to a *gfp* reporter had shown that the *hlh-17* reporter construct is expressed in the CEP sheath (CEPsh) glia cells and unidentified motor neurons in the ventral nerve cord, while the *hlh-32* reporter fusion was expressed in an unidentified pair of head neurons; no expression could be discerned for the *hlh-31* promoter fusion construct [55,60]. Promoter fusion constructs can miss *cis-*regulatory elements and, particularly in the case of the *hlh-17/31/32* locus, the genomic rearrangements leading to the duplication of the ancestral gene may have resulted in complex arrangements of *cis-*regulatory elements. To eliminate these concerns, we tagged each of the 3 endogenous loci with *gfp*, using CRISPR/Cas9 genome engineering. The *gfp* tagged loci show expression patterns that are indeed different from the previously reported promoter fusions. We find that HLH-17::GFP and HLH-32::GFP protein are exclusively detectable in 3 neuron classes, the bilaterally symmetric glutamatergic AUA interneuron class and 2 B-class cholinergic ventral cord motor neurons, DB2 and VB2 (**Fig 2**). Together with a few other head and neck muscle-innervating motor neurons, these motor neurons are part of the retrovesicular ganglion (RVG), which is located at the anterior end of the ventral nerve cord. DB2 and VB2 are the most anteriorly located B-type motor neurons in the RVG and have lineage histories that are distinct from other DB and VB class members [61,62]. Based on scRNA-seq data [19,63], DB2 and VB2 are also molecularly subtly distinct from other DB and VB class members. Due to their location, the RVG motor neurons are analogous to the branchial motor neurons in the brain stem of vertebrates, but little is known about how RVG motor neuron subtypes are made to become different from other motor neurons of the same class.

Expression of HLH-17::GFP and HLH-32::GFP in AUA, DB2, and VB2 is observed throughout all larval and adult stages and commences when those neurons are generated (**Fig 2**). Its postembryonic expression is consistent with scRNA-seq data [19] (**Fig 1A**). Notably, however, we detected no HLH-17::GFP protein expression in CEPsh glia (or any other glia) at any developmental stage, indicating that *hlh-17* transcripts, detected by scRNA-seq analysis [19] and inferred from transcriptional reporter constructs [55,60] may not become translated into detectable protein levels in the CEPsh glia. We have occasionally observed similar transcript and protein discordance for homeobox genes [12,19].

In contrast to HLH-32 and HLH-17, expression of HLH-31::GFP, encoded by the gene that directly neighbors HLH-17 (and which displays a somewhat degenerate bHLH domain sequence; **S1 Fig**), was not detectable in our analysis at any stage in any cell within or outside the nervous system. scRNA transcriptome analysis detects transcripts for *hlh-31* in the DB2 and VB2 neurons (like its neighboring *hlh-17* gene), and also in the CAN neurons [19] (**Fig 1A**). However, as with *hlh-17* transcripts in the CEPsh cells (also detected by scRNAseq), no readily detectable protein appears to be made from these *hlh-31* transcripts.

### Removal of *hlh-17*, *hlh-31*, and *hlh-32* does not obviously affect glia differentiation

To assess potential functions of *hlh-17*, *hlh-31*, and *hlh-32*, we generated single, double, and triple mutant strains using CRISPR/Cas9 genome engineering. Unlike the previously generated *hlh-17*, *hlh-31*, and *hlh-32* mutant alleles, which left parts of each gene intact [55], our engineered alleles each deleted the entire respective locus (**Fig 1C**). Due to overlaps in gene expression, and the sequence identity of the bHLH domains (**S1B Fig**), we considered possible functional redundancies and therefore started our analysis by examining *hlh-17/hlh-31/hlh-32* triple null mutant animals. These were generated in 2 consecutive genome editing rounds, first removing the 2 adjacent *hlh-17* and *hlh-31* loci (*syb6635)* and then removing the more distal *hlh-32* locus (*syb6773)* in the background of the *syb6635* allele. From here on, we refer to these *hlh-32(syb6773)hlh-17hlh-31(syb6635)* triple null mutants as “*hlh-17/31/32*^*null*^*”*. These triple null mutant animals are viable, fertile and display no obvious morphological or behavioral abnormalities.

Consistent with the absence of HLH-17/31/32 protein expression in the CEPsh glia, we observed no effects of *hlh-17/31/32*^*null*^ mutants on CEPsh glia differentiation, as assessed by examination of CEPsh morphology and marker gene expression (**S2A and S2B Fig**). This is in line with a previous study that used partial deletions of the bHLH domains of *hlh-17/31/32*, displaying no effect on CEPsh morphology and marker expression [55].

### *hlh-17* and *hlh-32* are required for AUA neuron differentiation

**Fig 3:**
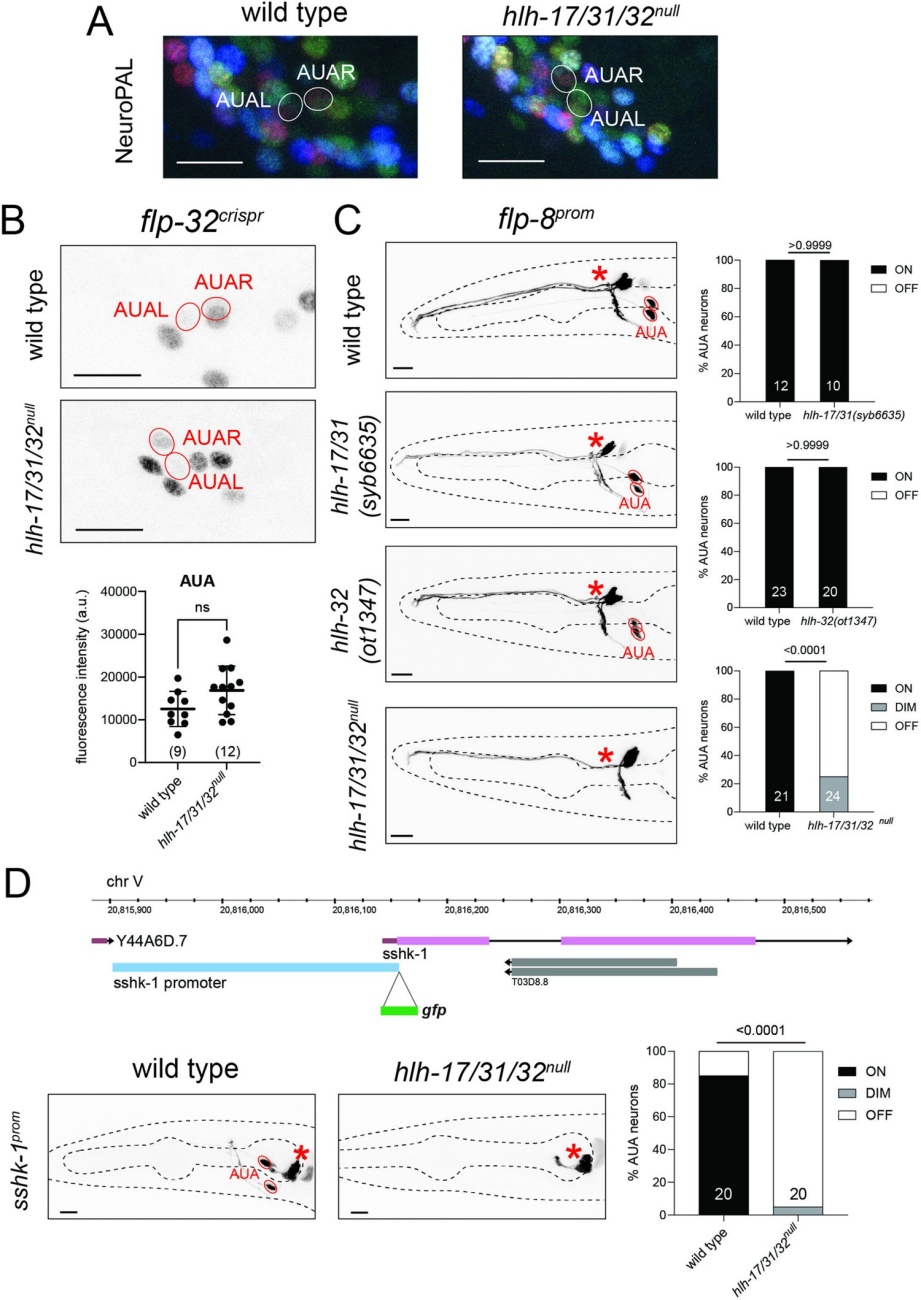
AUA cell fate analysis of *hlh-17*, *hlh-31*, and *hlh-32* mutant animals. (**A, B**) Triple *hlh-17/31/32*^null^ mutants do not show a change of NeuroPAL *(otIs669)* coloring of AUA (panel A) or expression of *flp-32(syb4374)* in AUA (panel B). In NeuroPAL, AUA neurons display a combination of mNeptune2.5 red pseudocolor driven by the *eat-4* promoter and a dim mTagBFP2 blue pseudocolor driven by the *mbr-1* promoter. In *hlh-17/31/32*^null^ mutants, NeuroPAL coloring of AUA is unchanged. Shown are representative images of wild-type and mutant animals with 10 μm scale bar. Statistical analysis was performed using unpaired *t* test. Individual points represent averages of the fluorescence intensities of the AUA neuron pair within an individual. The number of animals scored are enclosed in parentheses. Error bars indicate standard deviation of the mean. ns: not significant. (**C**) Expression of *flp-8* reporter *(ynIs78)* in AUA is affected only in *hlh-17/31/32*^null^ triple mutants. Individual *hlh-17/31(syb6635)* and *hlh-32(ot1347)* mutants do not show loss of the *flp-8* reporter. The *flp-8* reporter is also expressed in the URX neurons, indicated by the asterisks. Shown are representative images of wild-type and mutant animals with 10 μm scale bar. Number of animals scored are shown within each bar. *P*-values were calculated using Fisher’s exact test. (**D**) A novel fate reporter for AUA was generated by fusing the promoter directly upstream *sshk-1* with *gfp (otEx8199)*. *hlh-17/31/32*^null^ triple mutants lose expression of *sshk-1* reporter in AUA. The *sshk-1* reporter is also expressed in the pharyngeal gland cells, indicated by the asterisks. Representative images of wild-type and mutant animals are shown with 10 μm scale bar. Number of animals scored are shown within each bar. *P*-values were calculated using Fisher’s exact test. Raw data for panels B–D can be found in S1 Data.

To examine AUA neuron identity specification, we used the NeuroPAL transgene, in which this neuron is marked with 2 distinct fate markers, *eat-4/VGLUT* and *mbr-1* [34]. We find that the NeuroPAL color code of the AUA neuron pair is not obviously changed in *hlh-17/31/32*^*null*^ animals (**Fig 3A**). Examining other AUA markers, we first investigated expression of the neuropeptide-encoding *flp-8* and *flp-32* genes [19,64]. We found that *flp-8*, but not *flp-32*, expression is affected in triple null mutants (**Fig 3B and 3C**). Neither *hlh-32(ot1347)* single mutants nor *hlh-17hlh-31(syb6635)* double mutants recapitulated the *flp-8* expression defects (**Fig 3C**), indicating that AUA-expressed *hlh-32* and *hlh-17* indeed act redundantly to specify *flp-8* expression in AUA. This is not surprising given that both proteins are co-expressed and display an identical amino acid sequence of their bHLH domain.

We generated another identity marker for the AUA neuron by interrogating the CeNGEN scRNA atlas for transcripts with selective, strong expression in AUA. We considered the *T03D8*.*7* locus, which codes for a small secreted protein with a ShKT domain that, in other species, acts as a toxin against pathogens by inhibiting potassium channels [65]. We named this gene *sshk-1* (for small ShKT domain) and found that a transcriptional reporter that encompasses the entire intergenic region to its preceding gene is indeed expressed exclusively in AUA within the entire nervous system; outside the nervous system expression is also observed in the exocrine pharyngeal gland cells, consistent with this protein having a potentially protective function against pathogens (**Fig 3D**). We found that expression of the *sshk-1*^*prom*^ reporter transgene is strongly affected in the AUA (but not gland cells) of *hlh-17/31/32*^*null*^ mutant animals (**Fig 3D**). In those animals where residual *sshk-1*^*prom*^ expression was visible, AUA neurite morphology appeared normal.

### *hlh-17* and *hlh-32* diversify VB1/VB2 motor neuron subtype identity

We interrogated motor neuron identity specification using again *hlh-17/31/32*^*null*^ triple mutant animals because of (a) the redundant functions of co-expressed *hlh-32* and *hlh-17* we had revealed above in the AUA fate analysis; and (b) because of the theoretical possibility that even though *hlh-31* showed no expression in wild-type animals, it may be up-regulated in *hlh-32; hlh-17* double mutants. Between the motor neurons that express *hlh-17* and *hlh-32*, VB2 and DB2, we focused our analysis on VB2 because more identity markers are available for VB2 than for DB2.

**Fig 4:**
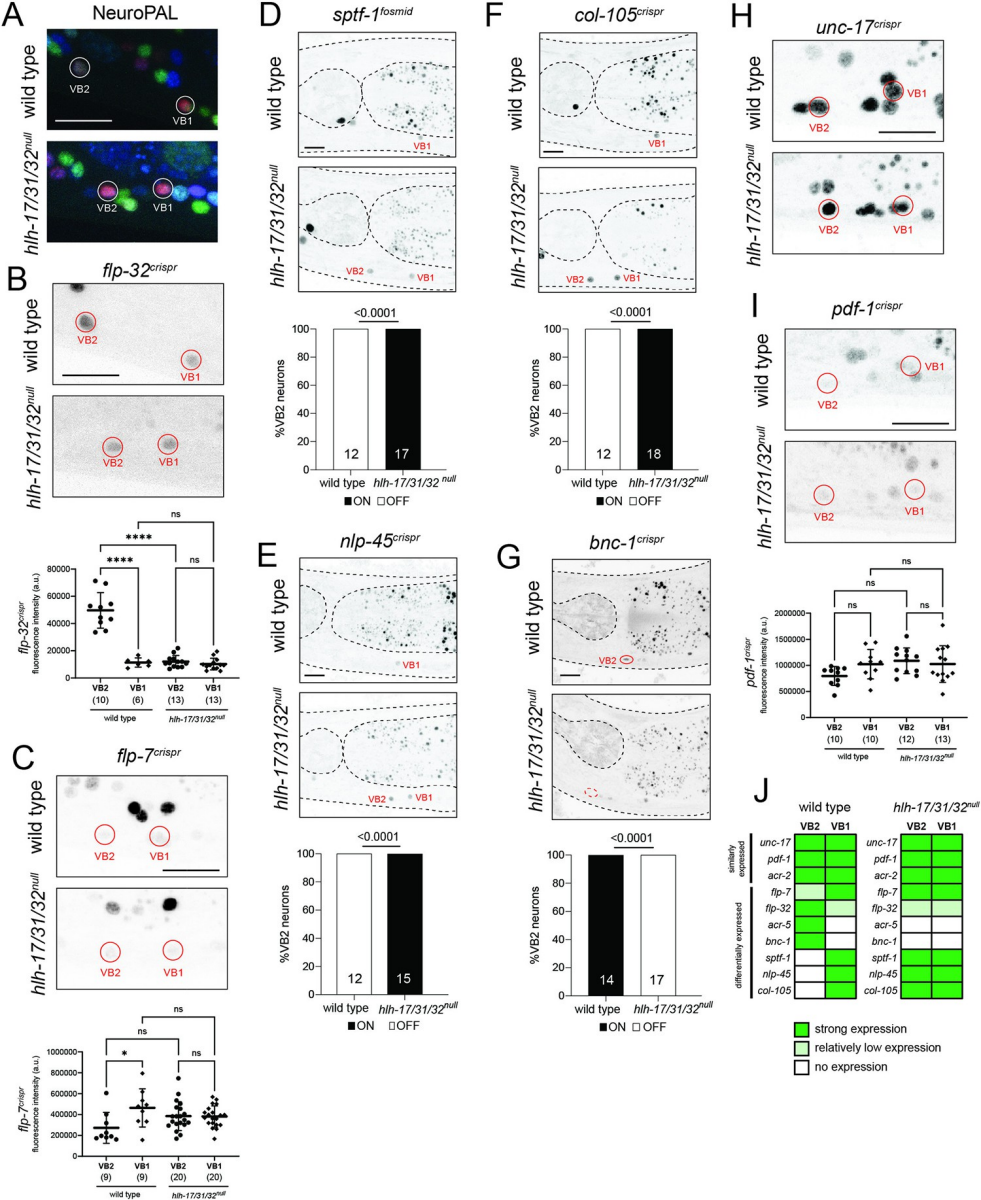
VB2 cell fate analysis of *hlh-17*, *hlh-31*, and *hlh-32* mutant animals. (**A**) In *hlh-17/31/32*^null^ mutants, VB2 adopts a VB1-like NeuroPAL *(otIs669)* coloring. (**B**) In *hlh-17/31/32*^null^ mutants, *flp-32(syb4374)* reporter allele expression in VB2 is decreased, reaching the same level of expression as that in VB1. (**C**) The difference in *flp-7(syb5413)* reporter allele expression levels between VB2 and VB1 is abolished in *hlh-17/31/32*^null^ mutants. (**D**) Expression of the *wgIs707* fosmid-based reporter for transcription factor *sptf-1* is normally restricted to VB1 but becomes derepressed in VB2 in *hlh-17/31/32*^null^ animals. (**E**) The *nlp-45(ot1032)* reporter allele marks VB1 alone in wild type but is ectopically expressed in VB2 in *hlh-17/31/32*^null^ animals. (**F**) The *col-105(syb6767)* reporter allele is a marker for VB1 that is ectopically expressed in VB2 in *hlh-17/31/32*^null^ animals. (**G**) The reporter allele for transcription factor *bnc-1(ot845)* loses expression in VB2 in *hlh-17/31/32*^null^ animals. (**H**) *hlh-17/31/32*^null^ mutants retain strong expression of *unc-17(syb4491)* in VB2 and VB1. (**I**) Expression of the *pdf-1(syb3330)* reporter allele in VB2 and VB1 remain unchanged in *hlh-17/31/32*^null^ mutants. (**J**) Schematic summarizing the relative expression of markers expressed in either VB2, VB1, or both, in wild-type and *hlh-17/31/32*^null^ animals. Differential expression of markers between VB2 and VB1 is abolished in *hlh-17/31/32*^null^ animals. The *acr-2* and *acr-5* reporters are part of the NeuroPAL transgene. Representative images of wild-type and mutant animals are shown with 10 μm scale bar. For panels D, E, and F, quantitative comparison of the fluorescence between VB2 and VB1 in *hlh-17/31/32*^null^ animals is in **S2C Fig**. For panels B, C, and I, statistical analysis was performed using one-way ANOVA with Tukey multiple comparisons test. Each point represents the corresponding neuron from one individual and the number of animals scored are enclosed in parentheses. Error bars indicate standard deviation of the mean. ****p ≤ 0.0001, *p ≤ 0.05, ns: not significant. For panels D–G, statistical analysis was performed using Fisher’s exact test, with the number of animals scored shown within each bar. Raw data for panels B–G and I can be found in S1 Data.

We first examined the effect of the *hlh-17/31/32*^*null*^ mutation on NeuroPAL, in which defects in neuronal specification can be detected through changes in neuron coloring conferred by specific sets of promoters [34]. We observed that in *hlh-17/31/32*^*null*^ animals, VB2 adopts NeuroPAL coloring that resembles another VB class member, VB1 (**Fig 4A**). The change in NeuroPAL coloring of VB2 can be attributed to loss of *acr-5* expression, which normally distinguishes VB2 from VB1 [34]. Previous cell fate marker analysis from our lab [34,66,67], as well as recent scRNA-seq analysis [19,63] revealed a number of additional genes that are differentially expressed in VB2 versus VB1, which allowed us to test whether *hlh-17/31/32* may indeed work to distinguish the identity of these neurons. We first interrogated markers shared by VB2 and VB1 but expressed at different levels, according to scRNA transcriptomes [19,63], namely the neuropeptides *flp-32* and *flp-7*. Using CRISPR/Cas9-engineered reporter alleles, we found that the difference in expression level of these markers that exists between VB2 and VB1 in wild-type animals is abolished in *hlh-17/31/32*^*null*^ mutants (**Fig 4B and 4C**).

If VB2 indeed acquires VB1 fate, markers exclusive to VB1 should become ectopically expressed in the VB2 neurons of *hlh-17/31/32*^*null*^ mutants. To examine such a potential VB2-to-VB1 transformation, we sought markers normally present only in VB1 but not VB2. A fosmid-based reporter transgene of the *sptf-1* zinc finger transcription factor had previously been shown to be expressed in VB1, but not VB2 [19] (**Fig 4D**). Moreover, we found that an *nlp-45* neuropeptide reporter allele, previously described to be in either VB1 or VB2 [67], is expressed in VB1, not VB2 (**Fig 4E**). In addition, based on scRNAseq data [19,63], we CRISPR/Cas9-engineered a reporter allele for the collagen *col-105*, and confirmed expression in VB1, not VB2 (**Fig 4F**). Armed with these 3 VB1(+) VB2(-) markers, we indeed found that all 3 (*sptf-1*, *nlp-45*, and *col-105*) become ectopically expressed in the VB2 neuron of *hlh-17/31/32*^*null*^ triple mutant animals at the same intensity as VB1 (**Figs 4D–4F and S2C**). Lastly, a zinc finger transcription factor, *bnc-1*, normally expressed in VB2 (and all other B-type motor neurons), but not VB1 [66], fails to be properly expressed in VB2 in *hlh-17/31/32*^*null*^ mutants (**Fig 4G**). In contrast, markers that are expressed at similar levels between VB2 and VB1, such as reporter alleles for the pan-cholinergic marker *unc-17/VAChT*, the *pdf-1* neuropeptide or the *acr-2* AChR, are unaffected in *hlh-17/31/32*^*null*^ mutants (**Fig 4H and 4I;** *acr-2* is located on the NeuroPAL transgene; **Fig 4A**). Taken together, *hlh-17/31/32* are neither required for the generation of VB2 nor for the adoption of B-motor neuron fate but differential gene expression that normally exists between VB2 and VB1 in wild-type animals is abolished in *hlh-17/31/32*^*null*^ animals, with VB2 taking on a VB1-like gene expression profile (**Fig 4J**). In other words, *hlh-17/31/32* instruct VB2 to become different from VB1 and an absence of these genes results in a homeotic neuronal identity transformation from VB2 to VB1, based on molecular markers (**Fig 4J**).

### Behavioral analysis of *hlh-17 hlh-31 hlh-32* triple mutant animals

*hlh-17* mutants were previously reported to display defects in the defecation motor program, which was ascribed to *hlh-17* function in the CEPsh glia [68]. We used our more unambiguous *hlh-17* null allele, in combination with the *hlh-31* and *hlh-32* null alleles, to avoid any potential issues of genetic redundancies among these genes, to confirm these defects. Testing both the posterior body wall contraction (pBOC) and the enteric muscle contraction (EMC) step of the defecation motor program, we observed no defects in *hlh-17/31/32*^*null*^ triple mutant animals (**S3A Fig**).

We also examined locomotory behavior of *hlh-17/31/32*^*null*^ mutant animals using an automated worm tracker system [35] and observed a number of defects, including in reversal behavior and speed (**S3B Fig**). We have not pursued the question of whether those defects are due to AUA and/or VB2 differentiation defects. We note, however, that whole brain activity recordings have shown striking correlations of the activity patterns of AUA with the command interneuron AVA [34], which controls several locomotory features that we find to be defective in *hlh-17/31/32*^*null*^ mutant animals.

### Exclusive expression of the PTF1a ortholog HLH-13 in the octopaminergic RIC interneurons

**Fig 5:**
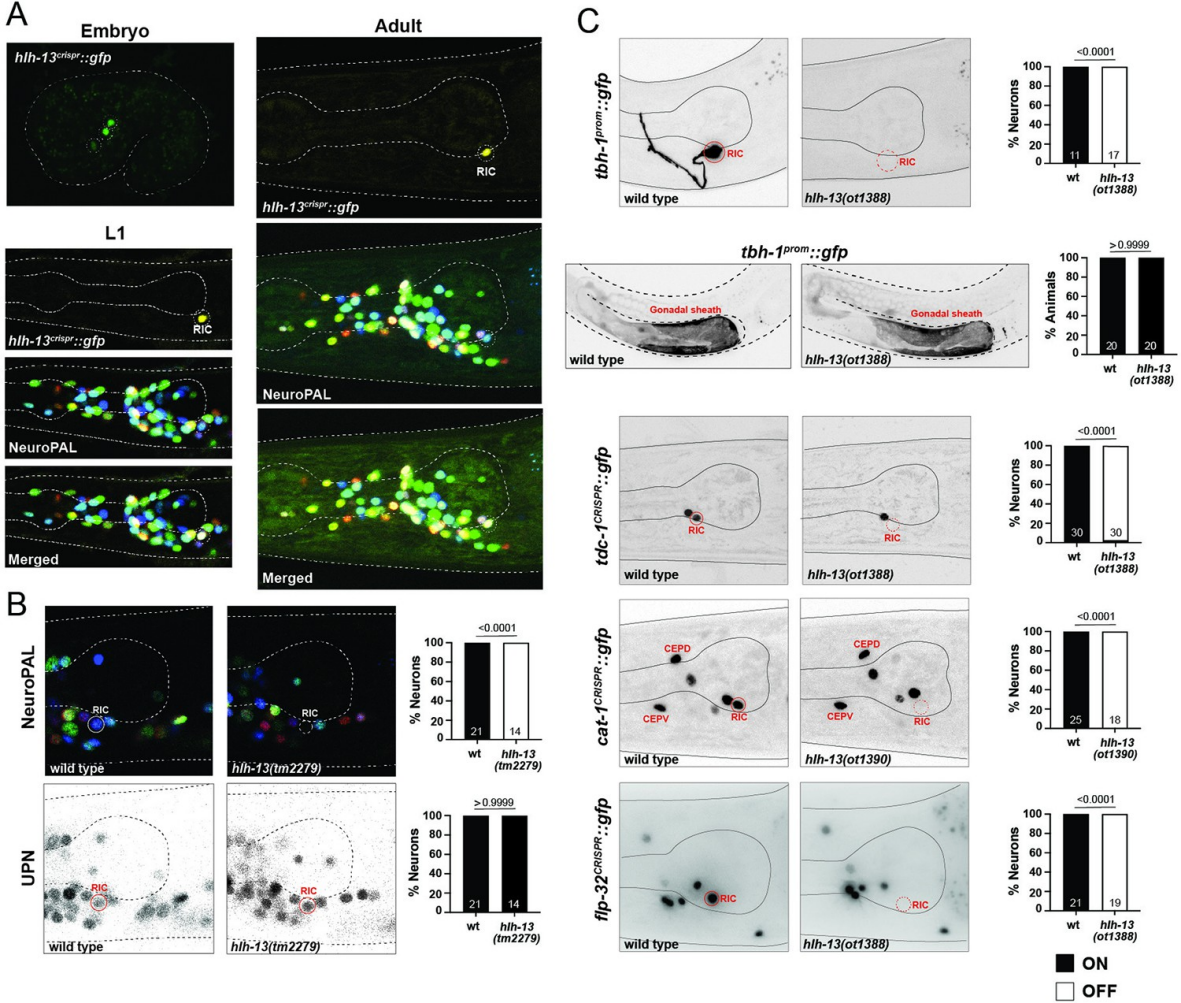
PTF1a and NATO3 ortholog HLH-13 is expressed in RIC and controls its differentiation. (**A**) Expression of the *hlh-13* CRISPR/Cas9-engineered reporter allele *syb7685* over the course of development. Overlay with the NeuroPAL transgene *otIs669* shows expression in RIC neurons. (**B**) Representative pictures and quantification showing loss of NeuroPAL (*otIs669*) coloring, but retention of panneuronal signal, in the RIC neurons of *hlh-13/PTF1a* mutants. Animals were scored at the young adult stage. Statistical analysis was performed using Fisher’s exact test. *N* is indicated within each bar and represents number of animals scored. (**C**) Representative pictures and quantification showing loss of RIC cell fate markers in *hlh-13/PTF1a* mutants. *tbh-1* expression in the gonadal sheath is unaffected by the loss of *hlh-13/PTF1a*. *cat-1* expression of the dopaminergic CEP neurons is not affected in *hlh-13/PTF1a* mutant animals. Reporter genes used are promoter fusion transgene for *tbh-1*(*nIs107*) and CRISPR/Cas9-engineered reporter alleles for *tdc-1*(*syb7768*), *cat-1*(*syb6486*), and *flp-32*(*syb4374*). Because *hlh-13* and *cat-1* are linked, the exact same mutation as *hlh-13(ot1388)* (**Fig 1C**) was generated in the background of the *cat-1(syb6486)* reporter; this allele is *hlh-13(ot1390)*. Animals were scored at the young adult stage. Statistical analysis was performed using Fisher’s exact test. *N* is indicated within each bar and represents number of animals scored. Raw data for panels B and C can be found in S1 Data.

The *C*. *elegans* genome encodes a single ortholog of the 2 paralogous vertebrate bHLH genes *PTF1a* and *NATO3* (aka FERD3L), called *hlh-13* [8,9,69] (**Fig 1B**). A previous analysis of *C*. *elegans hlh-13/PTF1a* suggested expression in dopamine neurons, based on a small reporter transgene that contained only 2.1 kilobases upstream of the gene [70,71]. However, the reported position of the GFP(+) cells in larval/adult animals is not fully consistent with expression in dopamine neurons and scRNA-seq data also suggests expression in, at most, a subset of dopaminergic neurons, plus other neurons that showed no expression of the previous reporter transgene [19]. To resolve these issues, we tagged the endogenous *hlh-13/PTF1a* gene locus with *gfp*, using CRISPR/Cas9 engineering (**Fig 1C**). Using the NeuroPAL cell ID tool [34], we observed strong HLH-13 protein expression exclusively in the sole octopaminergic neuron class in *C*. *elegans*, RIC [72], throughout all larval and adult stages (**Fig 5A**). This is consistent with scRNA-seq data of L4 stage animals [19] (**Fig 1A**). RIC is also among the very few neuron classes that continuously express the E/Da protein HLH-2 throughout postembryonic life (**Fig 1A**) [15,73]. Together with their documented capacity to interact in yeast 2-hybrid assays [48], this co-expression indicates that like its vertebrate ortholog [74], HLH-13/PTF1A may heterodimerize with the E protein HLH-2 in vivo.

In the embryo, we observe HLH-13 protein expression in 3 postmitotic neuron pairs that resolves to expression in a single neuron pair (RIC) before hatching (**Fig 5A**). Since *hlh-13/PTF1a* scRNA transcripts are observed in embryonic (but not postembryonic) CEP neurons [19,75], we crossed the *hlh-13/PTF1a* reporter allele with a transgenic line that expresses RFP in dopaminergic neurons including CEP, but a lack of overlap of the fluorescent signals ruled out that these neurons are the embryonic CEP neurons. Based on embryonic lineaging data of an *hlh-13/PTF1a* reporter transgene by the EPIC project [76], these HLH-13(+) cells may be the sister of the RIC neurons that are fated to die during embryogenesis and the 2 cousins of the RIC neurons, the SIBD neurons.

### Octopaminergic RIC neurons fail to properly differentiate in *hlh-13/PTF1a* mutant animals

We used 2 mutant alleles to analyze *hlh-13/PTF1a* function: (a) a deletion mutant, *tm2279*, generated by the National BioResource Project (NBRP) knockout consortium, which deletes most of the locus, including most of its bHLH domain; and (b) *ot1388*, a complete locus deletion that we generated by CRISPR/Cas9 genome engineering (**Fig 1C**). Animals carrying either allele are viable, fertile and display no obvious morphological abnormalities. We assessed the function of *hlh-13/PTF1a* in neuronal differentiation by again using the NeuroPAL transgene. The RIC neuron class, the only cell type with strong and consistent postembryonic expression, display a loss of both markers on the NeuroPAL transgene, *ggr-3* and *mbr-1* (**Fig 5B**). Persistence of the NeuroPAL panneuronal signal in the RIC neurons of *hlh-13/PTF1a* mutant animals demonstrates that the RIC neurons are still generated (**Fig 5B**).

We extended our analysis of the differentiation defects in RIC interneurons by examining additional markers of their octopaminergic identity, including the 2 biosynthetic enzymes TDC-1 and TBH-1 [72]. TDC-1, an aromatic amino acid decarboxylase, is exclusively expressed in the octopaminergic RIC and tyraminergic RIM neurons to convert tyrosine to tyramine and TBH-1, a tyramine hydroxylase, is exclusively expressed in RIC to convert tyramine to octopamine [72]. We found that expression of both a *tbh-1* reporter transgene and a *tdc-1* reporter allele [77] is eliminated in the RIC neurons of *hlh-13/PTF1a* mutants (**Fig 5C**). Moreover, a CRISPR/Cas9-engineered reporter allele of the monoaminergic vesicular transporter CAT-1/VMAT [77] also fails to be expressed in the RIC neurons of *hlh-13/PTF1a* mutant animals (**Fig 5C**). Expression of *cat-1/VMAT* is unaffected in dopaminergic neurons in *hlh-13/PTF1a* mutants (**Fig 5C**), consistent with *hlh-13/PTF1a* not being expressed in dopaminergic neurons.

The RIC neuron pair also expresses a unique signature of neuropeptide-encoding genes [19]. We used a CRISPR/Cas9-engineered reporter allele of one of them, *flp-32* [78], and found that its expression is eliminated in *hlh-13/PTF1a* null mutants (**Fig 5C**).

We examined the fate of other neurons that are predicted to express much lower levels of *hlh-13/PTF1a* mRNA based on embryonic or postembryonic scRNA-seq data, including all dopaminergic neurons, and found no differentiation defects using NeuroPAL. The abovementioned *flp-32* RIC marker is also expressed in ALA (in which *hlh-13* transcript but no HLH-13::GFP protein can be detected), but we observed no *flp-32* expression defects in ALA. We conclude that *hlh-13/PTF1a* selectively affects RIC neuron differentiation.

### Physiological and behavioral consequences of *hlh-13/PTF1a* loss

**Fig 6:**
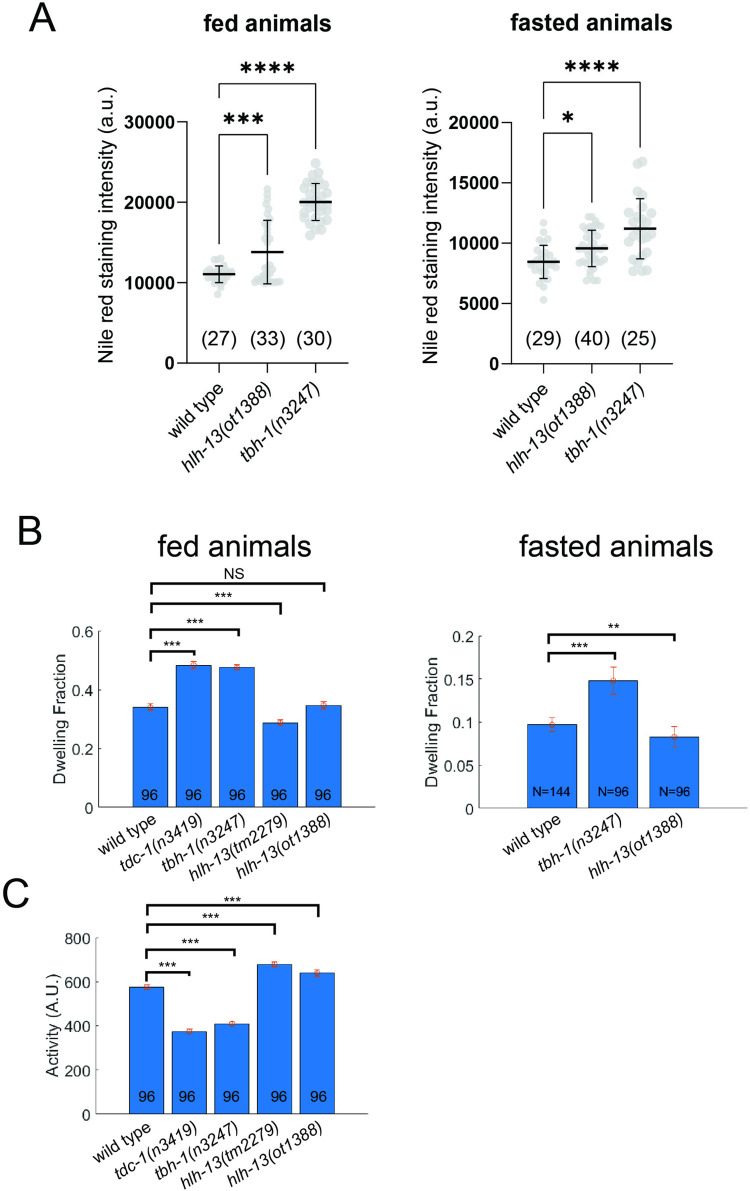
Physiological consequences of loss of the PTF1a and NATO3 ortholog *hlh-13*. (**A**) *hlh-13(ot1388)* and *tbh-1(n3247)* mutant animals display comparable nile red lipid staining defects in well-fed and in starved animals. Statistical analysis was performed using one-way ANOVA with Dunnett’s multiple comparisons test. Error bars indicate standard deviation of the mean. Number of animals scored for each genotype is enclosed in parentheses. ****p ≤ 0.0001, ***p ≤ 0.001, *p ≤ 0.05. (**B, C**) Two independent *hlh-13/PTF1a* mutant alleles do not phenocopy the previously reported [40] (and herein repeated) locomotory defects of *tbh-1(n3247)* mutant animals, indicating that the cellular source of octopamine required for proper locomotory behavior may not be the RIC neurons. Error bars indicate mean ± SEM. ***P < 0.001, **P < 0.01, ns: not significant, compared with wild-type, Dunnett’s multiple comparison tests. Raw data for panels A–C can be found in S1 Data.

The *hlh-13-*expressing RIC neuron pair is the only neuron class that produces octopamine [72], an endocrine monoaminergic regulator, analogous to vertebrate norepinephrine, that links nutrient cues to lipolysis to maintain energy homeostasis [79]. We confirmed that loss of *tbh-1* results in an up-regulation of lipid stores in the intestine, both under well-fed and starved conditions (**Fig 6A**), as previously reported [79]. Consistent with *hlh-13/PTF1a* controlling RIC differentiation, and hence octopamine synthesis and release, we find that *hlh-13/PTF1a* mutant animals also display improper fat accumulation in the intestine, both under well-fed and starved conditions (**Fig 6A**).

As another adaptation to changes in food availability, octopamine also controls dwelling behavior. Quantitative locomotory analysis of feeding *C*. *elegans* showed that *tbh-1* mutant animals exhibit a larger fraction of dwelling and smaller fraction of roaming compared to wild-type worms [40]. Interestingly, this difference is not recapitulated in *hlh-13/PTF1a* mutant animals (**Fig 6B and 6C**), which exhibit roaming and dwelling at rates similar to those of wild-type worms. Since octopamine is produced not only in the RIC neurons, but also in gonadal sheath cells [72], and since we find gonadal expression of *tbh-1* to be unaffected by loss of *hlh-13/PTF1a* (**Fig 5C**), we surmise that octopamine from the gonadal sheath cells may be sufficient for modulation of dwelling behavior.

### Exclusive expression of the NHLH1 and NHLH2 ortholog HLH-15 in 2 neuron classes

**Fig 7:**
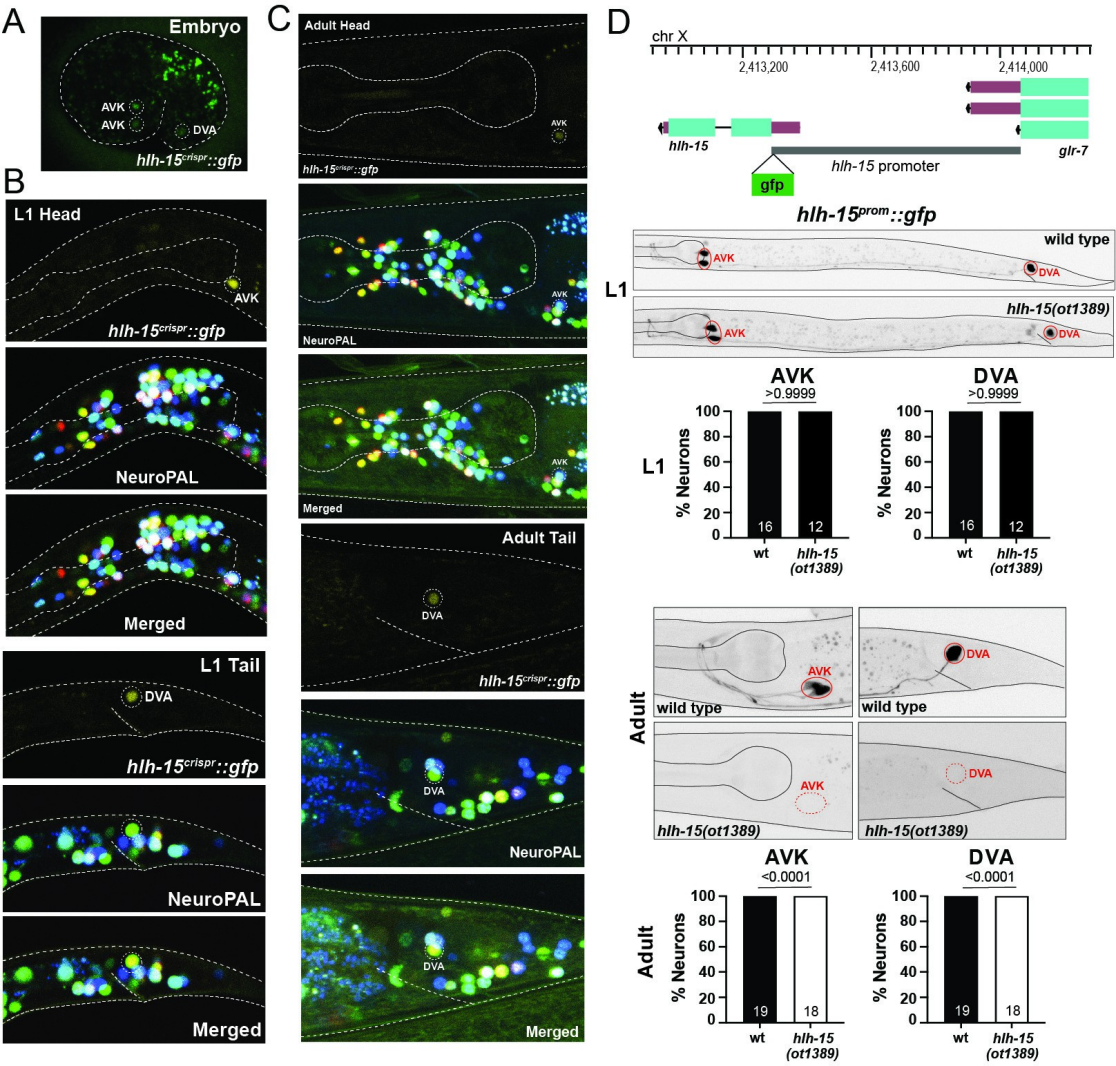
NHLH1 and NHLH2 ortholog HLH-15 is expressed in AVK and DVA neurons. (**A–C**) Expression of the *hlh-15/NHLH* CRISPR/Cas-9-engineered reporter allele *syb7688* over the course of development. Overlay with the NeuroPAL transgene *otIs669* shows expression in AVK and DVA neurons. (**D**) *hlh-15/NHLH* autoregulates its own expression in AVK and DVA neurons. Top panel shows *hlh-15* locus. The whole upstream intergenic region (from ATG to −777 bp) was fused to *gfp* to generate *wwEx42* [48]. Representative pictures and quantification showing expression of *hlh-15/NHLH* promoter fusion (*wwEx42*) in *hlh-15/NHLH* mutants. Animals were scored at the L1 (middle panel) and young adult stage (bottom panel). Statistical analysis was performed using Fisher’s exact test. *N* is indicated within each bar and represents number of animals scored. Raw data for panel D can be found in S1 Data.

Previous phylogenetic analysis has shown that the 2 paralogous vertebrate bHLH genes NSCL1/NHLH1/HEN1 and NSCL2/NHLH2/HEN2 have a single *C*. *elegans* ortholog, *hlh-15* [8,9,69] (**Fig 1B**). A promoter-based transgene of *hlh-15/NHLH* was previously reported to be expressed in the tail DVA neuron and either the RIF or RIG head interneurons [48]. We used the CRISPR/Cas9 system to tag the *hlh-15/NHLH* locus with *gfp* (**Fig 1C**) and again used the NeuroPAL cell ID tool to assess the sites of *hlh-15*::*gfp* expression [34]. Throughout all postembryonic stages and adulthood, we observe HLH-15/NHLH protein expression exclusively in 3 neurons, the 2 peptidergic bilateral AVK interneurons AVKL and AVKR as well as the unpaired cholinergic tail interneuron DVA (**Fig 7A–7C**). The exclusive expression in these 2 neuron classes commences at around the comma/1.5-fold stage and persists throughout all larval and adult stages. Both the embryonic and postembryonic reporter allele expression are consistent with scRNA-seq data from embryos and L4 stage animals [19,75] (**Fig 1A**). We re-examined the previously published promoter-reporter fusion construct for *hlh-15* and found that this construct is indeed also expressed in DVA and the AVK neurons (and not in RIF or RIG, as previously described; [48]). AVK and DVA are also among the very few neuron classes that continuously express the E/Da homolog HLH-2 throughout postembryonic life (**Fig 1A**) [15,73]. This indicates that HLH-15/NHLH may, like its vertebrate homolog [80], heterodimerize with the E protein HLH-2, a notion supported by HLH-15::HLH-2 protein interactions in yeast 2-hybrid assays [48].

Continuous expression of transcription factors throughout the life of a cell often reflect transcriptional autoregulation [81,82]. To ask whether *hlh-15/NHLH* autoregulates, we utilized a previously described transcriptional reporter of the *hlh-15/NHLH* locus in which its intergenic 5′ promoter region was fused to *gfp* [48]. This reporter recapitulates continuous expression in the AVK and DVA neurons (**Fig 7D**). In an *hlh-15/NHLH* null mutant background, expression of the reporter is initially normally observed in AVK and DVA, but it fades during postembryonic life to result in complete absence at the adult stage (**Fig 7D**). This observation demonstrates autoregulation of the *hlh-15/NHLH* locus.

### The peptidergic AVK neuron class fails to differentiate properly in *hlh-15/NHLH* mutant animals

We used 2 mutant alleles to analyze *hlh-15/NHLH* function: (a) a deletion mutant, *tm1824*, generated by the NBRP knockout consortium, which deletes most of the locus, including most of its bHLH domain; and (b) a complete locus deletion, *ot1389*, that we generated by CRISPR/Cas9 genome engineering (**Fig 1C**). Animals carrying either allele are fully viable and display no obvious morphological abnormalities. Unlike their vertebrate counterparts [28], *hlh-15/NHLH* mutants display no obvious fertility defects.

**Fig 8:**
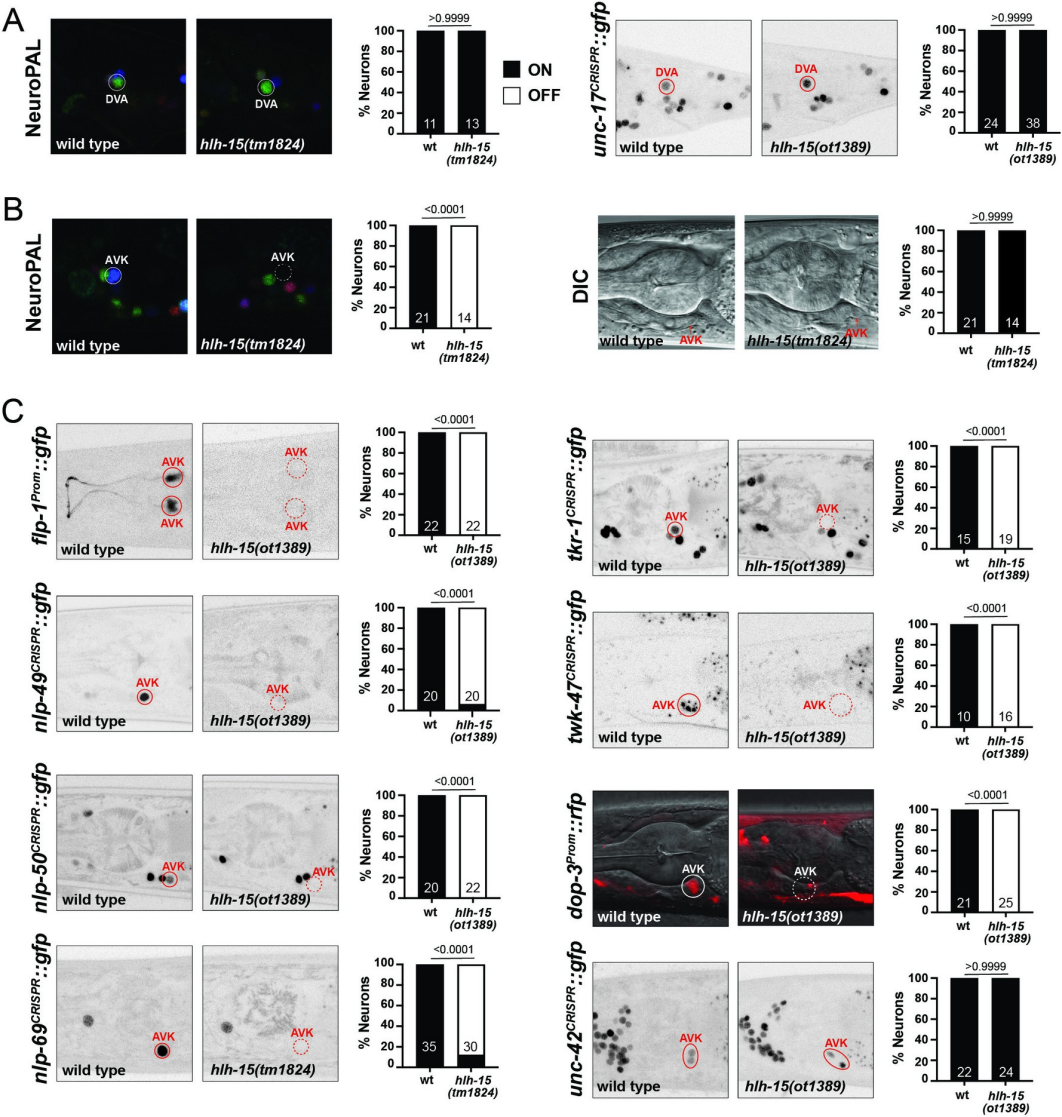
*hlh-15/NHLH* gene affects the differentiation of the AVK interneurons. (**A**) Representative pictures and quantification showing unaffected NeuroPAL (*otIs669*) coloring in DVA of *hlh-15/NHLH* mutants (left panel). The expression of the CRISPR/Cas9-engineered reporter allele *unc-17*(*syb4491*) in DVA is also not affected (right panel). (**B**) Representative pictures and quantification showing loss of NeuroPAL (*otIs669*) coloring in AVK (left panel) in *hlh-15/NHLH* mutants. Despite loss of NeuroPAL coloring, AVK is still generated in *hlh-15/NHLH* mutants, as visualized by DIC microscopy (right panel). (**C**) Representative pictures and quantification showing loss of cell fate markers in *hlh-15/NHLH* mutants. Reporter transgenes used are *flp-1*(*bwIs2*), *dop-3(vsIs33)*, and CRISPR/Cas9-engineered reporter alleles *nlp-49*(*syb3320*), *nlp-50*(*syb6148*), *nlp-69*(*syb4512*), *tkr-1*(*syb4595*), *twk-47*(*bab385*), and *unc-42*(*ot986*). Animals were scored at the young adult stage. Statistical analysis was performed using Fisher’s exact test. *N* is indicated within each bar and represents number of animals scored. Raw data for panels A–C can be found in S1 Data.

We assessed the potential function of *hlh-15/NHLH* in controlling fate specification of the AVK and DVA neurons by first using NeuroPAL, the marker transgene in which all neurons express specific codes of cell fate markers [34]. In NeuroPAL, the AVK neurons are marked with the *flp-1* neuropeptide gene and DVA is marked with the *nlp-12* neuropeptide and the 2 ionotropic glutamate receptors *nmr-1* and *glr-1*. NeuroPAL colors in *hlh-15/NHLH* mutants indicate a loss of the AVK color code (*flp-1* expression), but no effect on the color code of the DVA neuron (**Fig 8A and 8B**). We confirmed the proper differentiation of DVA in *hlh-15/NHLH* mutants using another DVA fate marker, a reporter allele for the acetylcholine vesicular transporter *unc-17/VAChT*, whose expression we found to be unaffected as well (**Fig 8A**).

We confirmed the AVK differentiation defects of *hlh-15/NHLH* null mutants using another transgene to measure *flp-1* expression, *bwIs2* [83] (**Fig 8C**). Given that AVK is a unique neuropeptidergic hub interneuron, receiving and sending a multitude of neuropeptidergic signals [84], we further probed the acquisition of AVK’s neuropeptidergic identity in *hlh-15/NHLH* mutant animals. Specifically, we probed the peptidergic identity of AVK by CRISRP/Cas9-engineered reporter alleles for 3 additional neuropeptides, predicted by scRNA-seq data to be expressed in AVK, *nlp-49*, *nlp-50*, and *nlp-69* [19]. *nlp-49* was previously also reported to be required in AVK to control motor behavior [33,85] and is, next to *flp-1*, the neuropeptide that is most selectively expressed in AVK [19]. We found that expression of *nlp-49* in AVK is strongly affected in *hlh-15/NHLH* mutant animals, while expression in other *nlp-49(+)* neurons is unaffected (**Fig 8C**). Similarly, expression of the *nlp-50* and *nlp-69* reporter alleles is also selectively lost in the AVK neurons of *hlh-15/NHLH* mutants (**Fig 8C**).

Apart from transmitting neuropeptidergic signals, AVK also expresses a multitude of neuropeptidergic receptors [19,84]. We examined one of them, the tachykinin receptor *tkr-1*, expressed in AVK and many other head neurons [84], and found its expression in AVK to be disrupted in *hlh-15/NHLH* mutant animals (**Fig 8C**). AVK also receives dopaminergic signals via the AVK-expressed dopamine receptor *dop-3* [41], and we find *dop-3* expression to also be disrupted in *hlh-15/NHLH* mutant animals (**Fig 8C**).

The nervous system-wide scRNA atlas of *C*. *elegans* predicts another unique identity marker of the AVK neurons, a 2 pore TWIK-type potassium channel, *twk-47* [19]. Its selective expression in AVK was recently validated with a promoter-based transgene construct [86] and is further validated with a CRISPR/Cas9-genome engineered, wrmScarlet-based reporter allele (kindly provided by T. Boulin). We find that expression of this *twk-47* reporter allele is also eliminated from the AVK neurons of *hlh-15/NHLH* mutants (**Fig 8C**).

Contrasting effects on neuron type-specific terminal “function” genes, we found that *hlh-15/NHLH* null mutant animals show normal expression of a previously described terminal selector of AVK differentiation, the *unc-42* homeobox gene [87,88], and also retain AVK as visualized by DIC microscopy (**Fig 8B and 8C**). These observations indicate that (a) *hlh-15/NHLH* is not acting as a proneural gene to determine the generation of the AVK neurons; and (b) that *hlh-15/NHLH* may work together, perhaps in a heteromeric, combinatorial manner, with *unc-42* as a terminal selector of AVK identity (see Discussion).

### Proprioceptive defects in *hlh-15/NHLH* mutant animals

**Fig 9:**
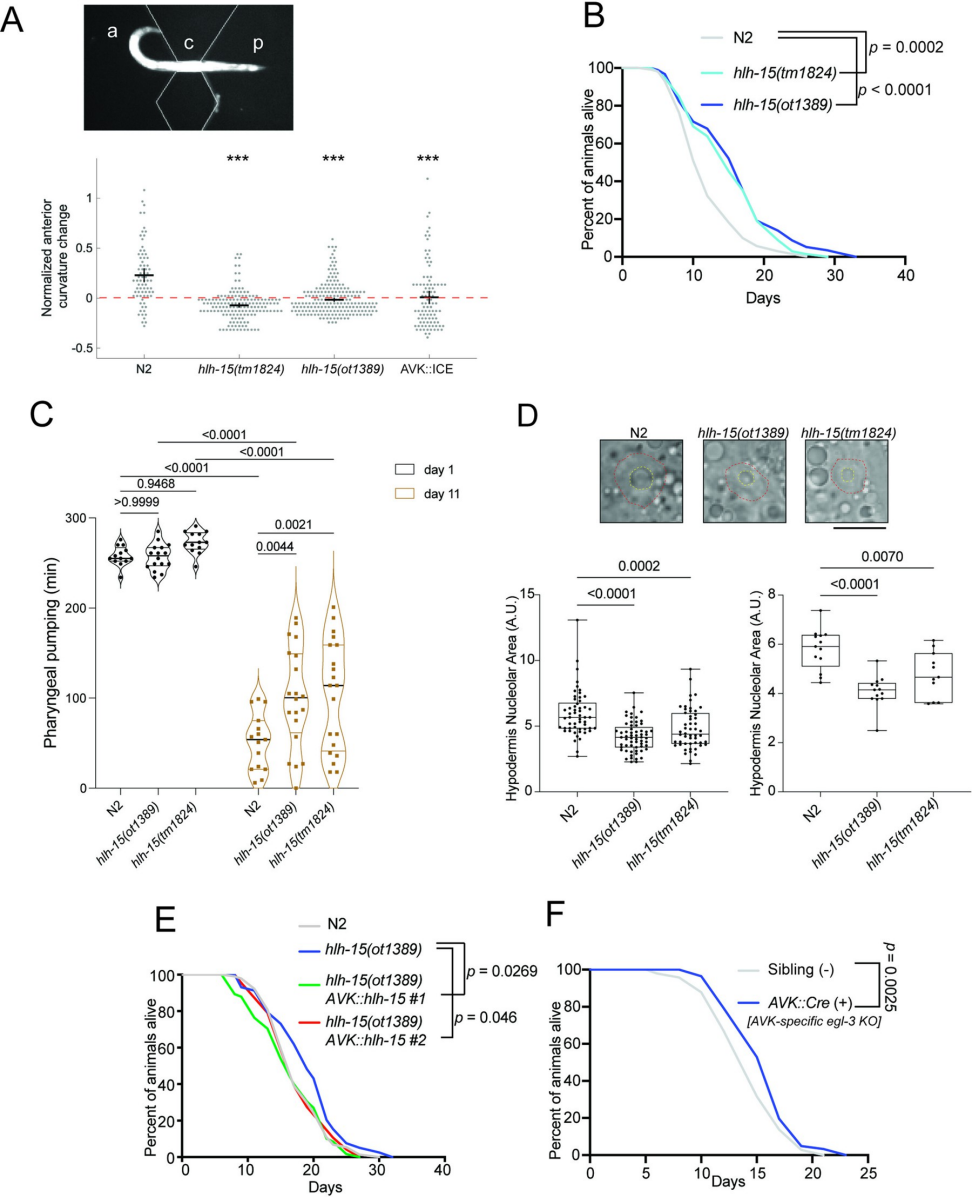
Behavioral and physiological analysis of *hlh-15/NHLH* mutants. (**A**) Measurement of the CCR. *C*. *elegans* partially constrained within a microfluidic channel [41]. a: anterior region, p: posterior region, c: constrained middle region. For each strain, the normalized anterior curvature change is equal to the anterior curvature amplitude during microfluidic constraint minus the anterior curvature amplitude of freely moving worms, divided by the anterior curvature amplitude of freely moving worms. *hlh-15(tm1824)* and *hlh-15(ot1389)* null mutants display defects in their CCR. These defects are similar to those observed upon genetic ablation of AVK using a transgenic array, *zxIs28[flp-1p(trc)*::*ICE; myo-2p*::*mCherry]*, in which the ICE caspase was driven by an AVK-specific *flp-1* promoter [33]. *N* ≥ 10 animals per condition. Error bars indicate mean ± SEM. ***P < 0.001, Dunnett’s multiple comparison tests. (**B**) *hlh-15(tm1824)* and *hlh-15(ot1389)* null mutants show increased lifespan. Survival plot of N2, *hlh-15(tm1824)*, and *hlh-15(ot1389)* animals. (**C**) *hlh-15/NHLH* null mutations attenuate the reduction in pharyngeal pumping rate associated with aging. Quantification of pharyngeal pumping rate for N2, *hlh-15(ot1389)*, and *hlh-15(tm1824)* at day 1 young adult and day 11 aged adult animals [45]. (**D**) Day 1 adult *hlh-15/NHLH* null mutants show reduced size of hypodermal cell nucleoli, a prospective hallmark of longevity. Representative DIC images of hypodermal nucleoli (yellow stippled lines; within nucleus, circled with red stippled line) and measurements of nucleolar size for N2, *hlh-15(ot1389)*, and *hlh-15(tm1824)* are shown with 10 μm scale bar. The quantification panel on the left shows the size of individual hypodermal nucleoli while the panel on the right shows average hypodermal nucleolar size for each animal. (**E**) Lifespan extension defects of *hlh-15(ot1389)* mutants are rescued by expressing *hlh-15/NHLH* selectively in the AVK neurons (*flp-1p*::*hlh-15)*. Survival plot of N2, *hlh-15(ot1389)*, and 2 independent transgenic lines (*otEx8247* and *otEx8248)* of *hlh-15(ot1389)* animals expressing genomic *hlh-15* DNA specifically in AVK. This assay could not be performed with the AVK::ICE lines because animals had a sick appearance. (**F**) Removal of neuropeptide processing in AVK neurons extends lifespan. Survival plot of floxed *egl-3(nu1711)* animals with the transgene (*kyEx6532*) or without the transgene (siblings) that expresses Cre recombinase specifically in AVK. For panels B, G, and H, statistics were performed using Log-Rank Test followed by Bonferroni corrections. *N* = 105 for each strain. All lifespan assays were replicated twice, with a repeat experiment for each shown in **S4 Fig**. Mean and 75% mortality lifespan tables can be found in **S2 Table**. For panel C, two-way ANOVA was performed, while for panels E and F, one-way ANOVA was performed, followed by Bonferroni correction. Raw data for panels A–F can be found in S1 Data. CCR, compensatory curvature response; DIC, differential interference contrast.

The AVK neurons were previously shown to act downstream of dopaminergic neurons to control proprioceptive behavior, a process mediated by the AVK-released and *hlh-15*-controlled *flp-1* gene [41]. Given that *hlh-15/NHLH* controls *flp-1* expression in AVK (the only *C*. *elegans* neuron that expresses *flp-1*), we predicted that *hlh-15/NHLH* mutants might display similar defects in proprioceptive behavior. We tested this prediction by measuring the AVK- and *flp-1-*dependent CCR in animals carrying either of the 2 available *hlh-15/NHLH* deletion alleles and found a severe reduction of the response, mimicking the genetic ablation of AVK (**Fig 9A**).

### *hlh-15/NHLH* acts in the AVK interneuron class to regulate life- and health span

RNAi knockdown of *hlh-15/NHLH*, as well as knockdown of its presumptive, class I heterodimerization partner *hlh-2*, has previously been reported to extend lifespan of *C*. *elegans* [89,90]. We found that the reported lifespan extension effects of *hlh-15(RNAi)* animals are recapitulated in animals that carry either of the 2 independent deletion alleles of the *hlh-15/NHLH* locus (**Fig 9B**). We extended the lifespan analysis of *hlh-15* mutants by considering health span of *hlh-15/NHLH* mutants. Health span can be measured by examining feeding behavior (pharyngeal pumping) in aged, but still alive worms [45]. We find this measure of health span to also be improved in both *hlh-15/NHLH* mutant strains (**Fig 9C**). Lastly, we tested a prospective hallmark of longevity, smaller nucleolar size [46] and found that in both mutant *hlh-15/NHLH* alleles, this parameter also shows the expected feature of long-lived animals (**Fig 9D**).

The previous RNAi-based analysis of *hlh-15/NHLH* [89] had not considered the potential cellular focus of action of *hlh-15/NHLH* for lifespan control. Since loss of *hlh-15/NHLH* only affects the differentiation of the AVK neurons, and not the only other neuron (DVA) in which *hlh-15/NHLH* is expressed, we tested the possibility that *hlh-15/NHLH* acts in AVK to control animal lifespan. Specifically, we asked whether the extended lifespan of *hlh-15/NHLH* is restored to normal wild-type–like lifespan by resupplying *hlh-15/NHLH* selectively in the AVK interneurons. We found that transgenic animals that re-express *hlh-15/NHLH* from an AVK-specific promoter in an *hlh-15/NHLH* null mutant background indeed displayed a rescue of the lifespan extension effect of *hlh-15* mutants (**Figs 9E and S4**), indicating that a single interneuron has the capacity to control animal lifespan.

Previous work has shown lifespan extension of animals in which the expression of an enzyme involved in branched-chain amino acid metabolism, *bcat-1* (branched-chain amino acid transferase-1), was reduced by RNAi [89]. Moreover, animal-wide RT-qPCR analysis suggested that levels of *bcat-1* transcripts are reduced in *hlh-15(RNAi)* animals [89]. A putative HLH-15/NHLH binding site in the *bcat-1* promoter made these authors suggest that HLH-15/NHLH directly controls *bcat-1* expression. We sought to independently probe the potential link of *hlh-15/NHLH* and *bcat-1* with alternative approaches. To this end, we first tagged the *bcat-1* locus with *gfp* using CRISPR/Cas9 genome engineering. As expected from a metabolic enzyme, we found very broad (albeit not entirely ubiquitous) and robust expression of *bcat-1* across all major tissue types (**S5 Fig**). However, in an *hlh-15/NHLH* null mutant background, *bcat-1* expression appeared unchanged, in both young and aged adult animals (**S5 Fig**). While this experiment does not entirely rule out that the lifespan extending effects of *hlh-15/NHLH* may be mediated by *bcat-1*, it indicates that *hlh-15/NHLH* and AVK may act through other means to control animal lifespan.

One set of possible lifespan modulating effector genes of *hlh-15/NHLH* are neuropeptides secreted by AVK. Based on scRNA-seq data, as well as reporter gene data, AVK shows highly selective expression of at least 8 neuropeptide-encoding genes, some of which are deeply conserved throughout animal phylogeny [19,85,91,92]. As demonstrated above, the expression of at least some, if not all AVK-expressed neuropeptides is under *hlh-15/NHLH* control (**Fig 8C**). Rather than venturing into testing lifespan effects of individual neuropeptide-encoding genes, we eliminated all neuropeptide processing selectively in the AVK neurons, using a conditional, floxed allele of the *egl-3* proprotein convertase required for neuropeptide processing [93,94]. We found that animals in which we removed *egl-3* in AVK also showed an increased lifespan (**Fig 9F**). We conclude that the lifespan-extending effect of *hlh-15/NHLH* can be explained via *hlh-15/NHLH* controlling the neuropeptidergic phenotype of AVK and, more generally, that a single interneuron class has the capacity to influence the lifespan of an animal.

## Discussion

### Revising expression patterns of conserved bHLH proteins in *C*. *elegans*

We have discovered functions of several, phylogenetically conserved bHLH proteins during terminal differentiation of postmitotic neurons, thereby expanding our understanding of bHLH gene function in the nervous system. The 3 clustered bHLH genes *hlh-17/31/32* were originally considered orthologs of Olig genes, which are important regulators of glia and motor neuron differentiation in vertebrates [95–97]. However, by sequence homology, HLH-17, HLH-31, and HLH-32 are the orthologs of the vertebrate bHLHe22 and bHLHe23 proteins rather than the closely related vertebrate Olig proteins. Unlike the vertebrate Olig proteins, vertebrate bHLHe22/e23 have no reported function in glia cells, but rather act in neuronal differentiation in select neuron classes in the retina, telencephalon, and spinal cord [20–26,59]. The *Drosophila* bHLHe22/e23 homolog also functions in motor neurons rather than glia [59]. Similarly, we find here that the *C*. *elegans* bHLHe22/e23 orthologs HLH-17 and HLH-32 are expressed and function in neurons, including motor neurons, but not glia. We cannot exclude that HLH-17/31/32 proteins are expressed below the levels of reporter allele-based detectability in the CEPsh glia cells. However, given the robust transcription of the *hlh-17/31/32* genes in CEPsh glia (based on scRNA-seq and promoter-fusion reporters), we consider posttranscriptional repression the most parsimonious explanation. Such posttranscriptional regulation is evident upon comparison of mRNA transcripts and homeodomain protein expression in several distinct neuron types [12,19].

We also found that the expression pattern of a reporter allele of the *PTF1a* homolog *hlh-13* is different from the previously reported expression of a multicopy *hlh-13/PTF1a* reporter transgene [70,71]. However, in this case, we do not infer posttranscriptional regulation as a source for differences, since our reporter allele data is consistent with scRNA-seq data. We rather consider previous reporter genes to either produce incorrect expression and/or the sites of expression have been incorrectly identified, a problem remedied by our usage of the neuronal landmark strain NeuroPAL.

Lastly, NeuroPAL has also been instrumental in defining the proper identity of *hlh-15/NHLH* expressing cells, which had only been incompletely achieved with previous reporter transgenes [48]. Taken together, our expression pattern analysis of these 5 conserved bHLH genes illustrated the importance of using the best possible tools (reporter alleles and cell ID tools) to properly infer protein expression patterns.

### Function of bHLH genes in neuronal identity specification

We discovered terminal neuronal differentiation defects for each of the bHLH subfamilies that we examined in this study. The *bHLHe22/e23* orthologs *hlh-17* and *hlh-32* function as “terminal subtype selectors” that diversify motor neuron subclass identity in the retrovesicular ganglion that harbors neurons akin to vertebrate branchial motor neurons. Loss of these factors results in a homeotic subtype identity transformation akin to those observed in *C*. *elegans* HOX mutants at the posterior end of the ventral nerve cord [98,99]. In another neuron class, AUA, which is potentially also involved in motor control [34], *hlh-17* and *hlh-32* affect select, but not all aspects of the differentiation program. The function of *hlh-17* and *hlh-32* in motor neuron differentiation is reminiscent of the function of *Drosophila* and vertebrate homologs of these genes, which play a role in various aspects of motor neuron specification [59,95,100,101], but we added here some intriguing granular detail to such motor neuron function. While anatomical reconstructions [102] and, more recently, scRNA-seq analysis [19,63], have revealed that the most anteriorly located B-type motor neurons within the retrovesicular ganglion are morphologically and molecularly distinct from other B-type motor neurons along the ventral nerve cord, no molecular mechanisms for their subclass diversification have been previously identified. We found that *hlh-17* and *hlh-32* do not affect the expression of features shared by B-type neuron types, but instead promote the expression of features that are characteristic of one subtype (VB2), while repressing features characteristic of another subtype (VB1). Generally, all motor neuron markers, including B-type motor neuron markers and at least some, if not all subtype-specific markers described here, are directly controlled by the terminal selector *unc-3*, an EBF-type transcription factor [66,103]. Akin to other subtype diversifiers (e.g., BNC-1) [66], we propose that HLH-17/32 act as subtype diversifiers to either directly or indirectly promote or antagonize the ability of UNC-3 to turn on select target genes.

*hlh-13/PTF1a* and *hlh-15/NHLH* appear to act in a canonical terminal selector-type manner in 2 different neuron classes, *hlh-13/PTF1a* in the octopaminergic RIC interneuron class and *hlh-15/NHLH* in the peptidergic AVK interneuron class. In both neuron classes, loss of the respective bHLH gene affects the expression of all markers tested, but they do not affect the generation of the neurons and they are continuously expressed throughout the life of the neuron to possibly maintain their identity. Such continuous expression is likely ensured by transcriptional autoregulation, as we have explicitly shown for *hlh-15/NHLH*.

The terminal selector roles of *hlh-13/PTF1a* and *hlh-15/NHLH* appear similar to the role of the ASC-type *hlh-4* bHLH as a validated terminal selector of the ADL sensory neuron class, where HLH-4 likely operates in conjunction with the common heterodimerization partner, the E/Da-homolog HLH-2, to co-regulate scores of terminal effector genes through direct binding to E-box motifs [17]. In both the AVK and RIC neuron classes, the HLH-15/NHLH and HLH-13/PTF1A protein, respectively, are also likely to heterodimerize with the common class I E/Da HLH-2 protein, based on: (a) co-expression of HLH-13/PTF1A and HLH-15/NHLH with HLH-2, which is expressed in 8 neuron classes, including the RIC and AVK neurons (ADL, ASH, PHA, PHB, RIC, AVK, DVA, and PVN) [15,73]; (b) physical interaction of HLH-15::HLH-2 and HLH-13::HLH-2 observed in protein-protein interaction assays [48]; (c) similar interactions of the vertebrate homolog of HLH-13 (PTF1a) and HLH-15 (NHLH1/2) with vertebrate E-proteins [74,80]. *hlh-2* is not expressed in the *hlh-17/31/32-*expressing AUA, DB2, and VB2 neurons, but this is consistent with Olig-related proteins acting as strong homodimers [104]. It is notable that for some of the neurons that express the common E/Daughterless bHLH component HLH-2 postembryonically, no expression of other group A bHLH proteins is observed (**Fig 1A**). This indicates that in these neurons (e.g., ASH or PHB), *hlh-2* may act as a homodimer, as it does in other non-neuronal cells [105]. A role of a terminal selector HLH-2 homodimer is conceivable because E-box motifs are enriched in the ASH and PHB terminal gene batteries [19].

Vertebrate homologs of *hlh-13* and *hlh-15* may have maintained their function as regulators of neuronal differentiation. The vertebrate bHLH PTF1a and its paralogue NATO3 have multiple functions both early and late during neuronal development [27,29]. The vertebrate *hlh-15* homologs NHLH1 and NHLH2 control the proper differentiation, and perhaps also maintenance, of several peptidergic hormone-producing cells in the hypothalamus [28,106,107], reminiscent of the role of *hlh-15/NHLH* in controlling the identity of the peptidergic AVK neurons. Roles in initiating and maintaining terminal differentiation programs as putative terminal selectors have, however, not yet been explicitly investigated for those vertebrate homologs, something that should be tested through temporally controlled removal of vertebrate homologs specifically during or after terminal differentiation.

In conclusion, based on our mutant and expression analysis, the bHLH genes described here do not act as neural fate-determining classic proneuronal bHLH genes in progenitors, but rather act as drivers of terminal differentiation programs in postmitotic neurons.

### Discovery of a lifespan-controlling peptidergic hub neuron

The very restricted expression of *hlh-15/NHLH* in exclusively 2 neuron classes, combined with its striking function in controlling animal lifespan led us to discover a function of a single interneuron class, AVK, in controlling lifespan of the animal. The impact of the *C*. *elegans* nervous system on animal lifespan has long been appreciated [108,109], but has largely been restricted to the sensory periphery, which releases distinct types of neuropeptides, mostly insulins, but also other types of neuropeptides to modulate animal aging [110–112]. We discover here a different type of neuron that controls lifespan, the AVK interneurons.

The AVK neuron class has been shown to regulate responses to various physiological states to control locomotion and behavior [33,41,85,93,113], but AVK has not previously been implicated in lifespan control. Moreover, AVK displays fundamental differences to neurons previously implicated in lifespan control. It is neither a sensory neuron, nor a main postsynaptic target of sensory neurons [102]. Its expression of a large number of neuropeptide receptors as well as of neuropeptides that act through receptors distributed throughout the animal nervous system has revealed AVK to be a central neuropeptidergic hub neuron [114] that likely controls internal states of the animal. We hypothesize that AVK may serve as an integrator of various internal states, resulting in the release of neuropeptide(s) that limit animal lifespan. It will be interesting to determine whether the release of these neuropeptides occurs continuously or only at specific time points during the life of the organism.

Both in terms of its peptidergic nature and also its developmental specification, AVK is conceptually remarkably similar to peptidergic neurons in the mammalian hypothalamus. Peptidergic hypothalamic neurons control internal states of mammals and have been implicated in lifespan control as well [115–117]. Strikingly, the mammalian homologs of the AVK-specifying *hlh-15* gene, *NHLH1* and *NHLH2*, control the proper specification of several classes of peptidergic neurons of the hypothalamus [28,106,107]. It will be intriguing to define with better resolution the exact type of hypothalamic neurons that continuously express and require NHLH1/2 during adulthood as this may help to more precisely determine—in analogy to HLH-15/NHLH expression and function—the nature of the neurons that may control lifespan in mammals. Intriguingly, the presently completely uncharacterized sole homolog of *NHLH1/2* and *hlh-15* in *Drosophila*, the *HLH4C* gene, appears to be expressed in the pars intercerebralis, which is considered to a hypothalamus-like structure in *Drosophila* [118].

### bHLH genes and homeobox gene as regulators of neuronal identity

The bHLH proteins that we have characterized here are unlikely to act in isolation but can rather be expected to act in combination with other transcription factors to control terminal neuron differentiation as terminal selectors. Their most likely partners are homeodomain transcription factors. Each neuron class in *C*. *elegans* is defined by a unique combination of homeodomain proteins and mutant analysis has corroborated their widespread deployment as drivers of terminal neuron differentiation programs [12,13]. Based on previous mutant analysis, HLH-15/NHLH (likely in combination with HLH-2) may cooperate with the Prop1-type homeodomain protein UNC-42 in the AVK neurons, where UNC-42 was previously shown to display similar differentiation defects to those that we have described here [83,88]. Similarly, in the octopaminergic RIC neurons, HLH-13/PTF1A likely acts together with the Pbx-type homeodomain protein UNC-62, whose loss also results in RIC differentiation defects [13]. And even though *hlh-17/31/32* may only control aspects of AUA differentiation, a potential cofactor is yet another homeobox gene, the POU homeobox gene *ceh-6*, which controls AUA differentiation [119]. Direct partnerships between bHLH and homeodomain proteins have been observed in other systems as well [120–122].

Taking a step back and comparing the relative functional deployment of the 42 bHLH family members and 102 homeobox genes in inducing and maintaining terminal differentiation programs in *C*. *elegans*, it is apparent that homeobox genes cover the differentiating nervous system much more broadly than bHLH factors do. Mutant analyses of many bHLH and homeobox genes support the notion that with notable exceptions (such as those described here), bHLH genes tend to be more prominently involved in early patterning events, while homeobox genes appear to be biased toward controlling terminal differentiation events in the nervous system. It will be fascinating to see whether this is an evolutionary conserved theme.

## Supporting information

S1 Fig Molecular phylogeny of the Olig/bHLHb4/b5 subfamily. (**A**) Phylogenetic relationship of *C*. *elegans* and human Olig/bHLHe22/e23 members. Tree generated at phylogeny.fr [123] with default parameters. (**B**) Protein sequence alignment of Olig/bHLHe22/e23 members across phylogeny. Created at phylogeny.fr by MUSCLE. Similar residues are colored as the most conserved one (according to BLOSUM62). Colors indicate average BLOSUM62 score: blue 1.5, low 0.5. (**C**) Tabular DiOPT scores of Olig/bHLHe22/e23 members as provided by MARRVEL [56]. One NeuroD homolog is provided as an “outgroup”. (**D**) *hlh-32*, *hlh-17*, and *hlh-31* are located close to each other in a region of *C*. *elegans* chromosome IV that is replete with small RNAs. From the genome browser of WormBase.
(TIF)

S2 Fig *hlh-17/31/32*^*null*^ animals show no measurable effects on CEPsh glia but display homeotic transformation of VB2 motor neurons into a VB1-like fate. (**A**) *hlh-17/31/32*^*null*^ animals show no defects in in the expression of the CEPsh marker *irIs67 (hlh-17prom*::*gfp)*. CEPsh morphology is also unaffected. Representative images of wild-type and mutant animals are shown with 10 μm scale bar. Number of animals scored are within each bar. *P*-values were calculated using Fisher’s exact test. (**B**) *hlh-17/31/32*^*null*^ animals show no defects in the expression of glial marker *kcc-3(syb4430)*. Expression in CEPsh is still clearly identifiable. Expression in other glia was also unaffected as indicated by counting the number of *kcc-3-*expressing cells. Representative images of wild-type and mutant animals are shown with 10 μm scale bar. Number of animals scored are shown within each bar. Statistical analysis for CEPsh expression was done using Fisher’s exact test, while that for counting *kcc-3*-expressing cells was performed using unpaired *t* test. Error bars for the scatter plot indicate standard deviation of the mean. (**C**) In wild-type animals, *sptf-1*, *col-105*, and *nlp-45* are expressed only in VB1 and not in VB2 (see Fig 4D–4F). In *hlh-17/31/32*^*null*^ animals, these VB1-only markers are ectopically expressed in VB2 in the same levels as VB1. Statistical analysis was performed using unpaired *t* test. Error bars indicate standard deviation of the mean. ns: not significant. Raw data for panels A–C can be found in S2 Data.
(TIF)

S3 Fig Behavioral analyses of *hlh-17/31/32*^*null*^ animals. (**A**) *hlh-17/31/32*^*null*^ animals do not display defects in the defecation motor program, contrary to a previous report using a different *hlh-17* single mutant allele [68]. Individual points represent measurements of individual worms. Statistical analysis was performed using unpaired *t* test. Error bars indicate standard deviation of the mean. ns: not significant. (**B**) Worm tracking of *hlh-17/31/32*^*null*^ animals reveal locomotory defects. Individual points represent measurements of individual worms. Data are pooled from 3 independent experiments. Statistical analysis was performed using unpaired *t* test. Error bars indicate standard deviation of the mean. ****p ≤ 0.0001, ***p ≤ 0.001, ns: not significant. *N* = 53 for wild type, *N* = 45 for *hlh-17/31/32*^*null*^. Raw data for panels A and B can be found in S2 Data.
(TIF)

S4 Fig Replication of *hlh-15/NHLH* lifespan assays. (**A**) Replication of the experiment in Fig 9B. (**B**) Replication of the experiment in Fig 9E. (**C**) Replication of the experiment in Fig 9F. Mean and 75% mortality lifespan tables can be found in **S2 Table**. Raw data for panels A–C can be found in S2 Data.
(TIF)

S5 Fig Loss of *hlh-15/NHLH* gene does not affect expression of *bcat-1* reporter allele. (**A**) Schematic of *bcat-1* locus showing insertion of *sl2*::*gfp*::*h2b* to generate *syb8612* reporter allele. (**B**) Representative pictures showing *bcat-1* expression in wild-type and *hlh-15/NHLH* mutants at day 1, day 4, and day 7 adult worms. Reporter gene used is CRISPR/Cas9-engineered reporter allele *bcat-1(syb8612)*. (**C**) Quantification of *bcat-1(syb8612)* expression in wild-type and *hlh-15/NHLH* mutants; all the head region was quantified. Animals were scored at day 1, day 4, and day 7 adult stage. Statistical analysis was performed using unpaired *t* test. (**D**) Representative pictures and quantification of *bcat-1(syb8612)* expression in AVK neurons in wild-type and *hlh-15/NHLH* mutants of day 1 adult worms. For day 4 and day 7 adult animals, AVK was difficult to identify through DIC and so AVK *bcat-1* expression for these animals was not scored. Statistical analysis was performed using unpaired *t* test. Raw data for panels C and D can be found in S2 Data.
(TIF)

S1 Table Strain list. (PDF)

S2 Table Lifespan analysis. (PDF)

S1 Data Collection of raw numerical data for Figs 3B–3D, 4B–4G, 4I, 5B, 5C, 6A–6C, 7D, 8A–8C and 9A–9F. (XLSX)

S2 Data Collection of raw numerical data for S2A–S2C, S3A, S3B, S4A–S4C, S5C and S5D Figs. (XLSX)

## Abbreviations

bHLH: basic helix-loop-helix
CCR: compensatory curvature response
DIC: differential interference contrast
EMC: enteric muscle contraction
NBRP: National BioResource Project
NR: Nile Red
pBOC: posterior body muscle contraction
RVG: retrovesicular ganglion
scRNA-seq: single-cell RNA sequencing
