# Supplementary figures and images for: Functional analysis of conserved C. elegans bHLH family members uncovers lifespan control by a peptidergic hub neuron
Source: PLoS Biol. 2025 Jan 6;23(1):e3002979. doi: 10.1371/journal.pbio.3002979 (PMC11703107; doi:10.1371/journal.pbio.3002979)

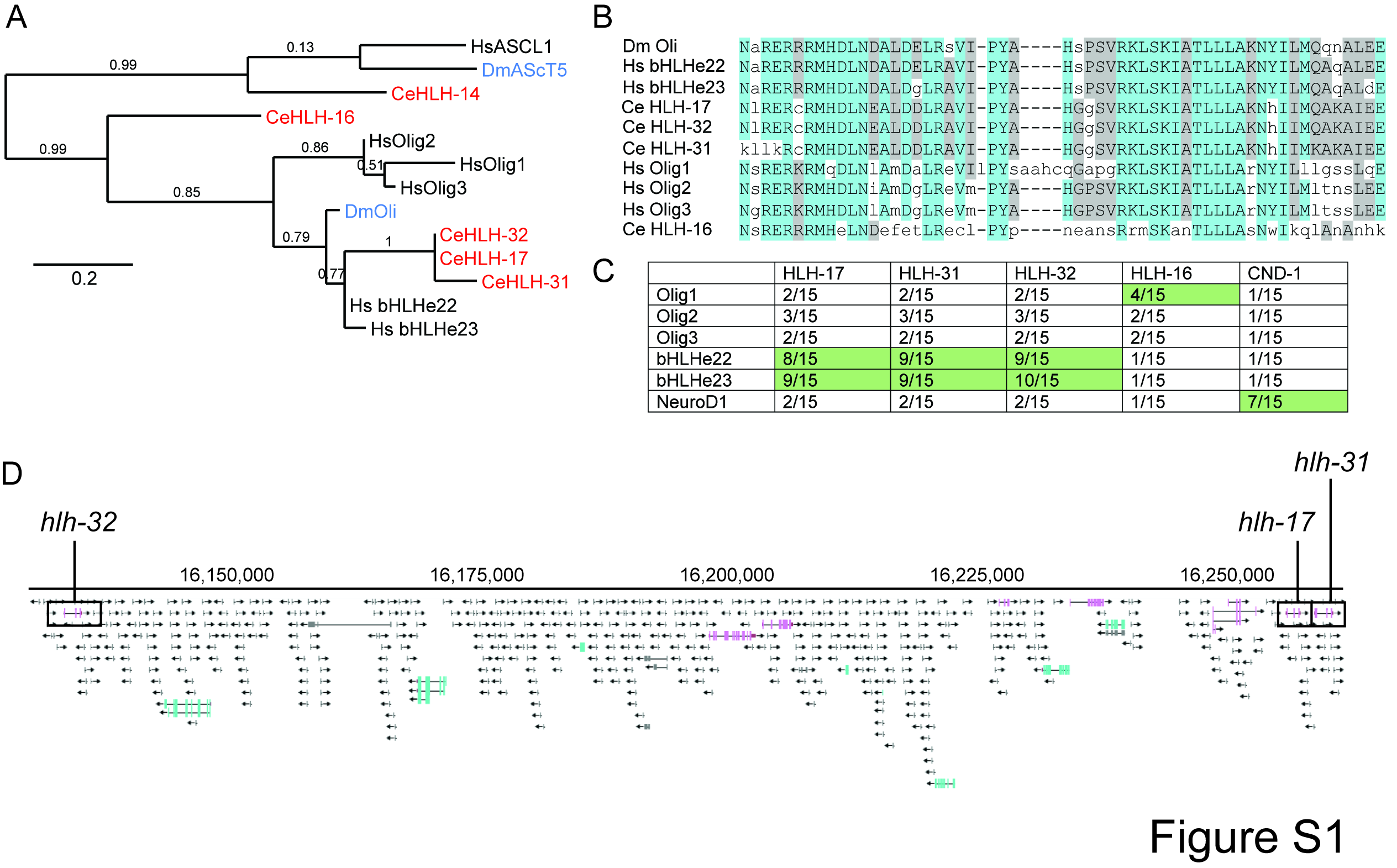

Supplement: S1 Fig — (A) Phylogenetic relationship of C. elegans and human Olig/bHLHe22/e23 members. Tree generated at phylogeny.fr [123] with default parameters. (B) Protein sequence alignment of Olig/bHLHe22/e23 members across phylogeny. Created at phylogeny.fr by MUSCLE. Similar residues are colored as the most conserved one (according to BLOSUM62). Colors indicate average BLOSUM62 score: blue 1.5, low 0.5. (C) Tabular DiOPT scores of Olig/bHLHe22/e23 members as provided by MARRVEL [56]. One NeuroD homolog is provided as an “outgroup”. (D) hlh-32, hlh-17, and hlh-31 are located close to each other in a region of C. elegans chromosome IV that is replete with small RNAs. From the genome browser of WormBase. (TIF) [file pbio.3002979.s001.tif]

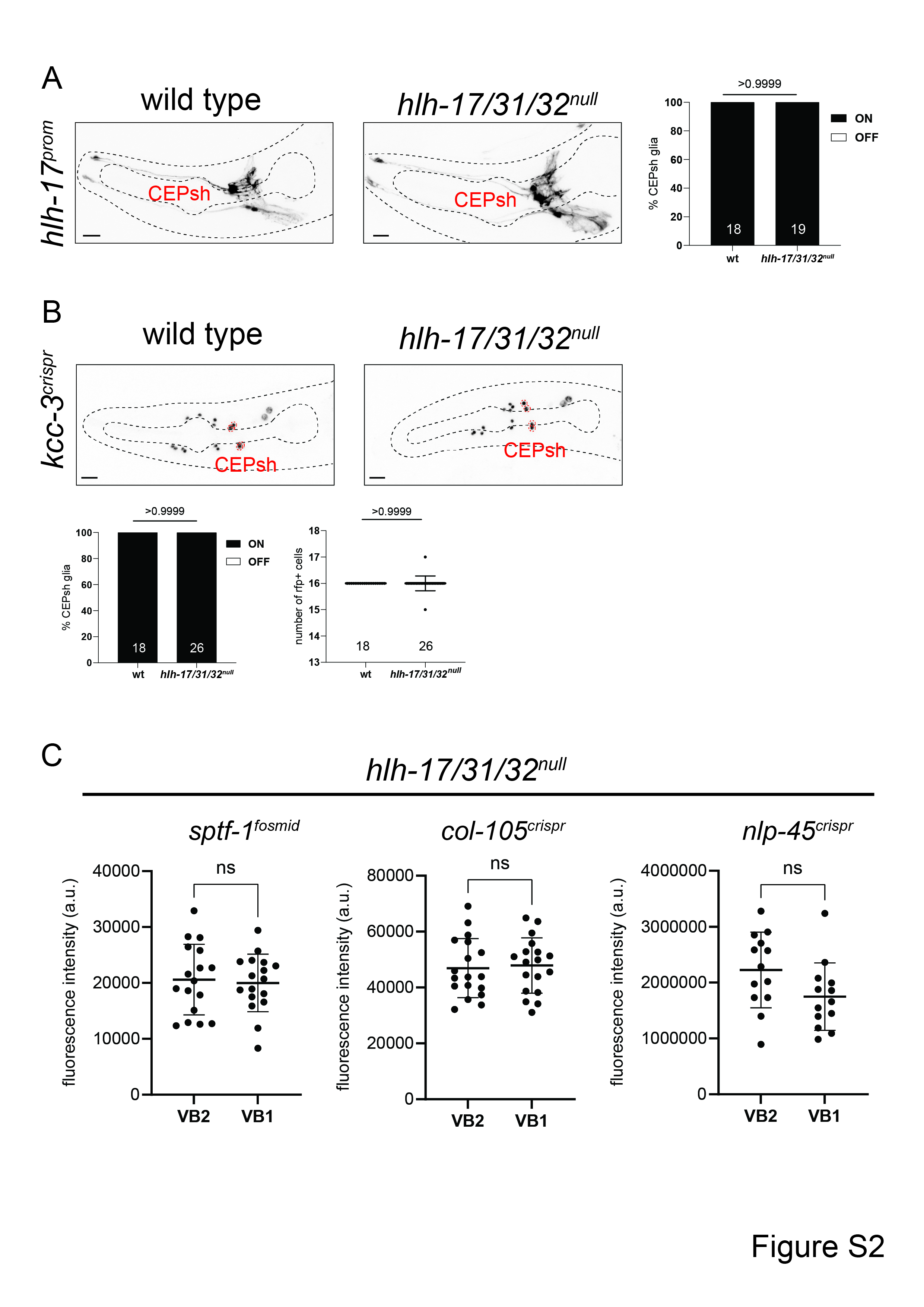

Supplement: S2 Fig — (A) hlh-17/31/32null animals show no defects in in the expression of the CEPsh marker irIs67 (hlh-17prom::gfp). CEPsh morphology is also unaffected. Representative images of wild-type and mutant animals are shown with 10 μm scale bar. Number of animals scored are within each bar. P-values were calculated using Fisher’s exact test. (B) hlh-17/31/32null animals show no defects in the expression of glial marker kcc-3(syb4430). Expression in CEPsh is still clearly identifiable. Expression in other glia was also unaffected as indicated by counting the number of kcc-3-expressing cells. Representative images of wild-type and mutant animals are shown with 10 μm scale bar. Number of animals scored are shown within each bar. Statistical analysis for CEPsh expression was done using Fisher’s exact test, while that for counting kcc-3-expressing cells was performed using unpaired t test. Error bars for the scatter plot indicate standard deviation of the mean. (C) In wild-type animals, sptf-1, col-105, and nlp-45 are expressed only in VB1 and not in VB2 (see Fig 4D–4F). In hlh-17/31/32null animals, these VB1-only markers are ectopically expressed in VB2 in the same levels as VB1. Statistical analysis was performed using unpaired t test. Error bars indicate standard deviation of the mean. ns: not significant. Raw data for panels A–C can be found in S2 Data. (TIF) [file pbio.3002979.s002.tif]

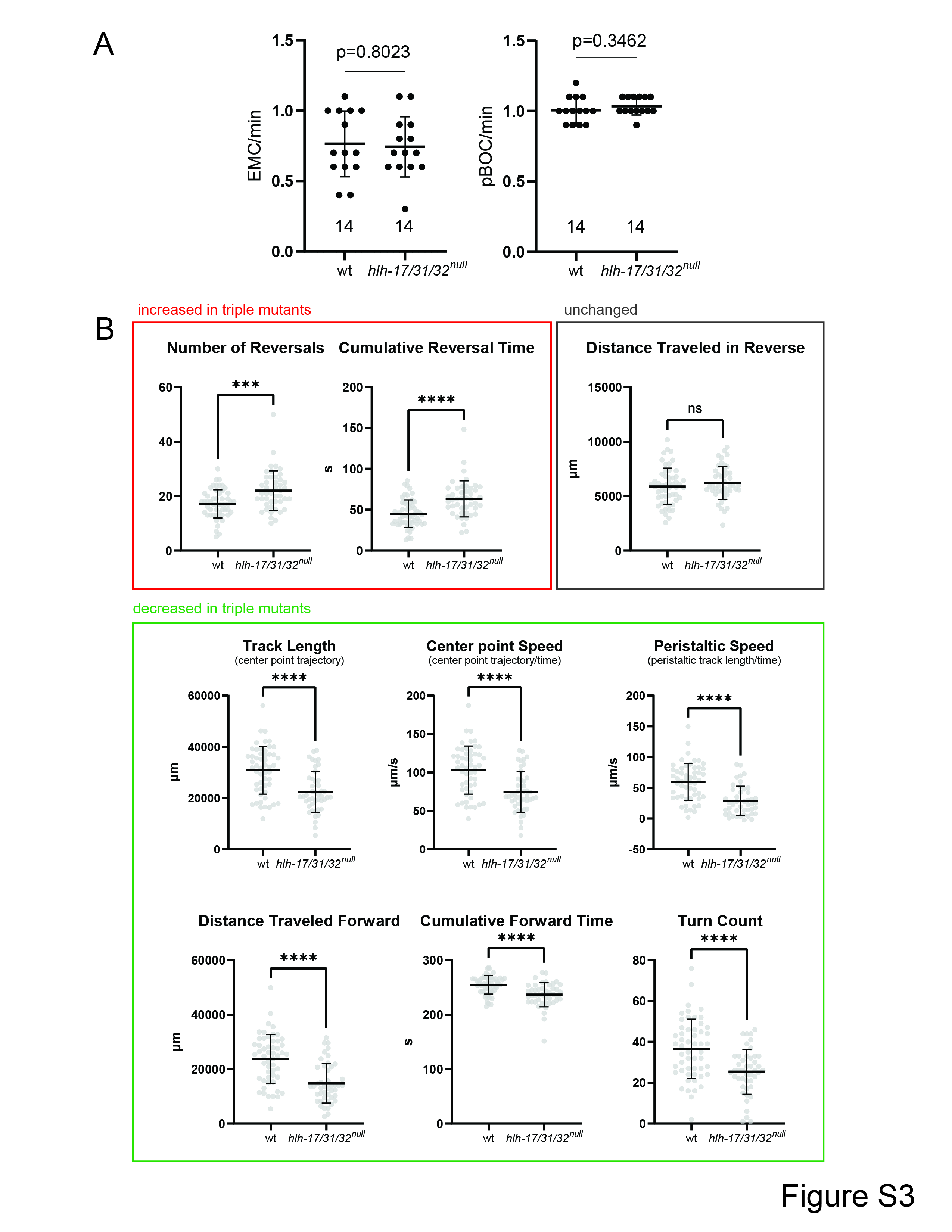

Supplement: S3 Fig — (A) hlh-17/31/32null animals do not display defects in the defecation motor program, contrary to a previous report using a different hlh-17 single mutant allele [68]. Individual points represent measurements of individual worms. Statistical analysis was performed using unpaired t test. Error bars indicate standard deviation of the mean. ns: not significant. (B) Worm tracking of hlh-17/31/32null animals reveal locomotory defects. Individual points represent measurements of individual worms. Data are pooled from 3 independent experiments. Statistical analysis was performed using unpaired t test. Error bars indicate standard deviation of the mean. ****p ≤ 0.0001, ***p ≤ 0.001, ns: not significant. N = 53 for wild type, N = 45 for hlh-17/31/32null. Raw data for panels A and B can be found in S2 Data. (TIF) [file pbio.3002979.s003.tif]

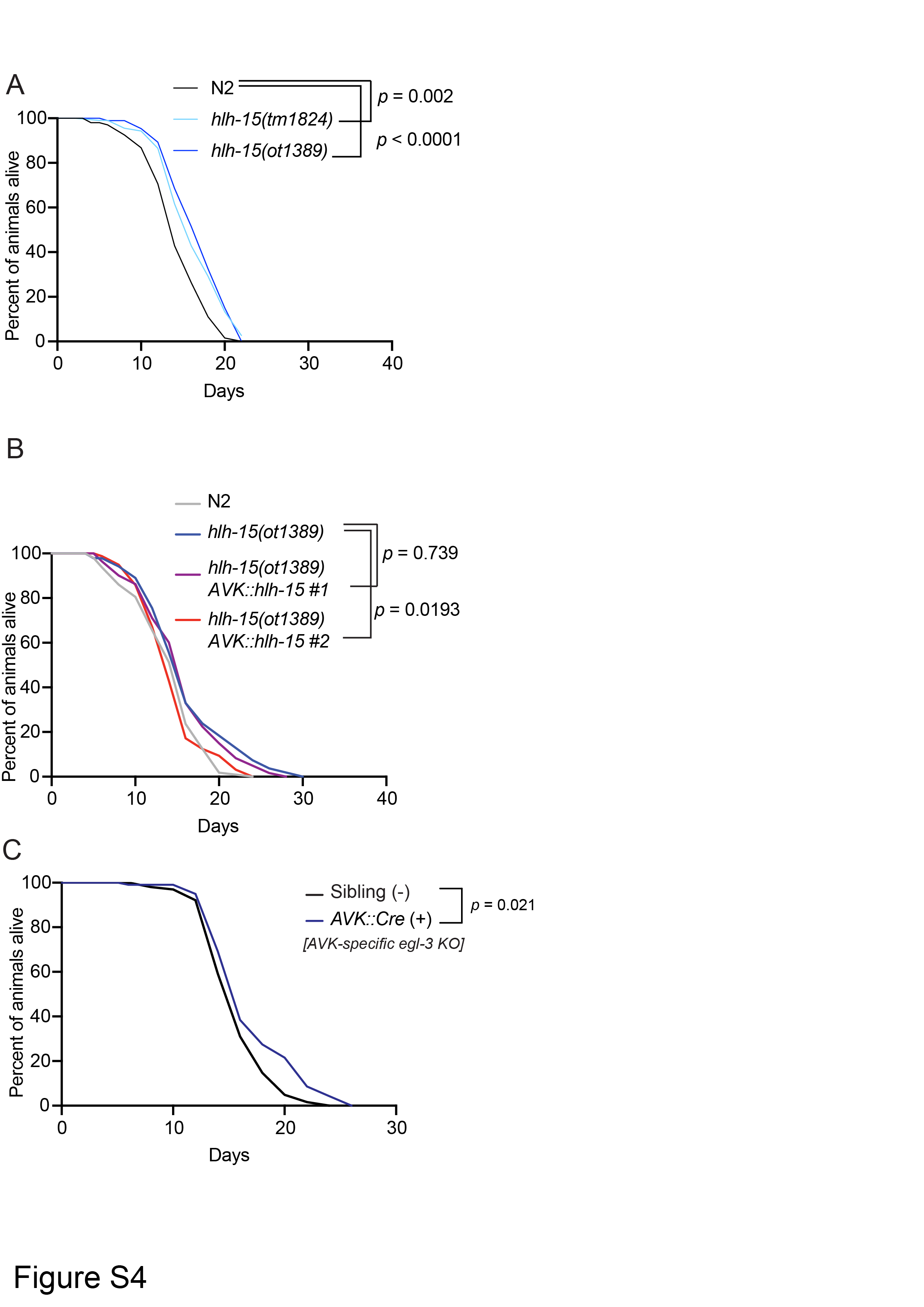

Supplement: S4 Fig — (A) Replication of the experiment in Fig 9B. (B) Replication of the experiment in Fig 9E. (C) Replication of the experiment in Fig 9F. Mean and 75% mortality lifespan tables can be found in S2 Table. Raw data for panels A–C can be found in S2 Data. (TIF) [file pbio.3002979.s004.tif]

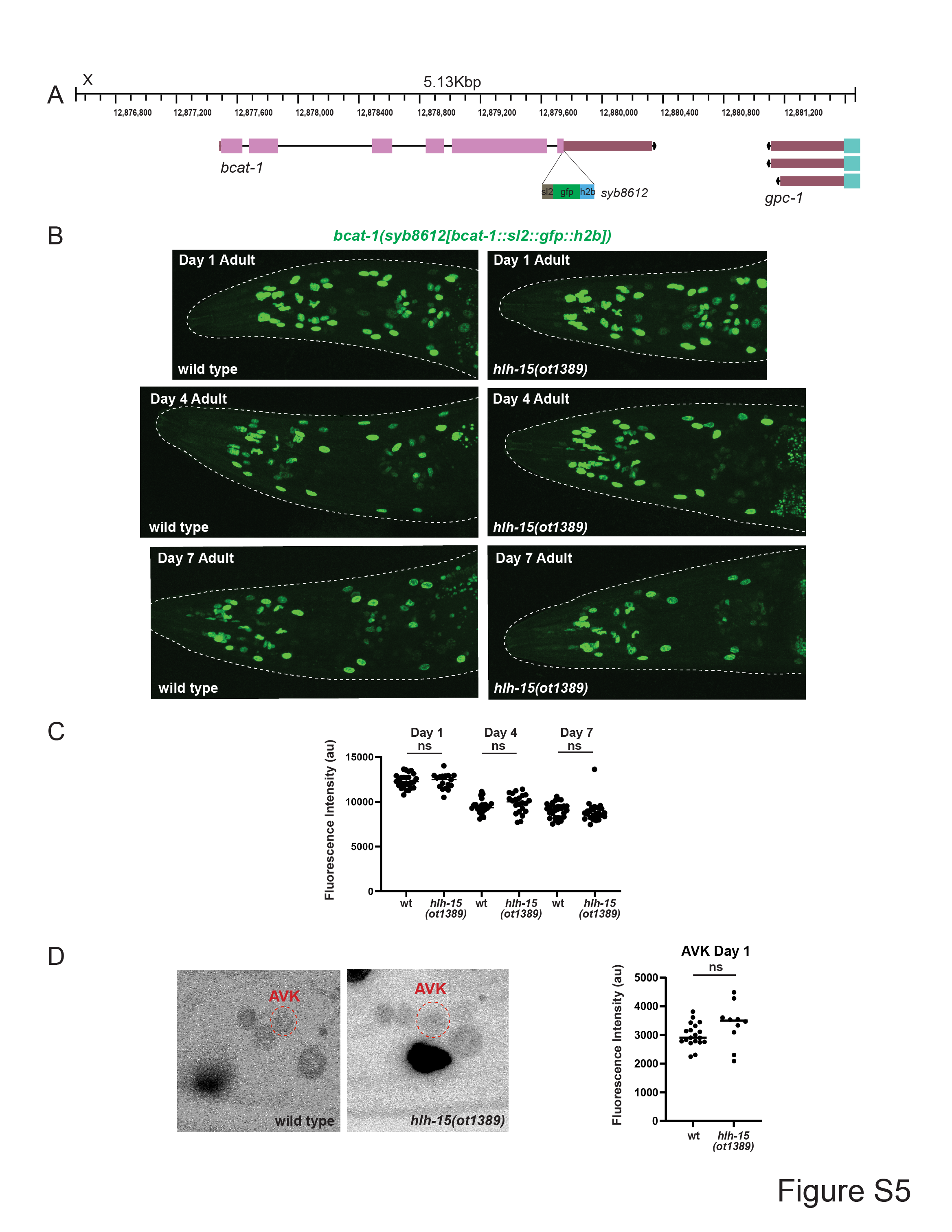

Supplement: S5 Fig — (A) Schematic of bcat-1 locus showing insertion of sl2::gfp::h2b to generate syb8612 reporter allele. (B) Representative pictures showing bcat-1 expression in wild-type and hlh-15/NHLH mutants at day 1, day 4, and day 7 adult worms. Reporter gene used is CRISPR/Cas9-engineered reporter allele bcat-1(syb8612). (C) Quantification of bcat-1(syb8612) expression in wild-type and hlh-15/NHLH mutants; all the head region was quantified. Animals were scored at day 1, day 4, and day 7 adult stage. Statistical analysis was performed using unpaired t test. (D) Representative pictures and quantification of bcat-1(syb8612) expression in AVK neurons in wild-type and hlh-15/NHLH mutants of day 1 adult worms. For day 4 and day 7 adult animals, AVK was difficult to identify through DIC and so AVK bcat-1 expression for these animals was not scored. Statistical analysis was performed using unpaired t test. Raw data for panels C and D can be found in S2 Data. (TIF) [file pbio.3002979.s005.tif]
