# Supplementary material for: Functional analysis of conserved C. elegans bHLH family members uncovers lifespan control by a peptidergic hub neuron
Source: PLoS Biol. 2025 Jan 6;23(1):e3002979. doi: 10.1371/journal.pbio.3002979 (PMC11703107; doi:10.1371/journal.pbio.3002979)
Supplement: S1 Table — (PDF) [file pbio.3002979.s006.pdf]

Table S1: Strain list

Strain Name	Genotype	Reference
**Mutants**		
PHX6635	*hlh-17hlh-31(syb6635) IV*	This study
PHX6773	*hlh-17hlh-31(syb6635)hlh-32(syb6773) IV*	This study
OH18840	*hlh-32(ot1347) IV*	This study
OH18614	*hlh-13(ot1388) X*	This study
OH18616	*hlh-13(ot1390) cat-1(syb6486) X; otIs669 V*	This study
OH18615	*hlh-15(ot1389) X*	This study
IU129	*hlh-13(tm2279) X*	NBRP
FX01824	*hlh-15(tm1824) X*	NBRP
MT9455	*tbh-1(n3247) X.*	PMID: 15848803
MT13113	*tdc-1(n3419) II.*	PMID: 15848803
**Transgenes**		
OH15363	*otIs669 (NeuroPAL) him-5(e1490) V*	PMID: 33378642
NY2078	*ynIs78[flp-8p::gfp] X*	PMID: 15236235
OH19054	*pha-1 (e2123) III; otEx8199 [sshk-1p::gfp, pha-1(+)]*	This study
VPR839	*unc-119(ed4) III; irIs67[hlh-17p::gfp + unc-119(+)]*	PMID: 24312354
OH19264	*hlh-15(ot1389) X; otEx8247[flp-1p::ghlh-15::SL2::tagRFP, ttx-3p::gfp]*	This study
OH19265	*hlh-15(ot1389) X; otEx8248[flp-1p::ghlh-15::SL2::tagRFP, ttx-3p::gfp]*	This study
ZX966	*zxIs28[pflp-1(trc)::ICE; pmyo-2::mCherry]*	PMID: 30392795
VL12	*unc-119(ed3) III; wwEx42[hlh-15p::GFP, unc-119(+)]*	PMID: 19632181
OH2007	*nIs107[tbh-1::gfp, lin-15(+] III*	PMID: 15848803
MU1085	*bwIs2[flp-1::GFP, rol-6(su1006)]*	PMID: 10648229
LX811	*vsIs33[dop-3::RFP] V; lin-15B&lin-15A(n765) X*	PMID: 15378064
OP707	*unc-119(tm4063) III; wgIs707[sptf-1::TY1::EGFP::3xFLAG + unc-119(+)].*	PMID: 16990816
CX18236	*egl-3 (nu1711) V; KyEx6532 [flp-1p(513 bp)::CRE (20 ng/uL) + elt-2p::nls::G*	PMID: 38573858
**Reporter alleles**		
PHX6303	*hlh-17(syb6303[hlh-17::gfp]) IV*	This study
PHX6112	*hlh-31(syb6112[hlh-31::gfp]) IV*	This study
PHX6078	*hlh-32(syb6078[hlh-32::gfp]) IV*	This study
PHX7685	*hlh-13(syb7685 [hlh-13::gfp]) X*	This study
PHX7688	*hlh-15(syb7688 [hlh-15::gfp]) X*	This study
PHX4430	*kcc-3(syb4430[kcc-3::sl2::TagRFP-T::h2b]) II*	This study
PHX3320	*nlp-49(syb3320[nlp-49::T2A::3XNLS::gfp]) X*	This study
PHX8612	*bcat-1(syb8612[bcat-1::SL2::GFP::H2B]) X*	This study
OH18694	*col-105(syb6767[col-105::sl2::gfp::h2b]) him-8(e1489) IV*	This study
OH19033	*pdf-1(syb3330[pdf-1::T2A::3XNLS::GFP]) III; otIs669 V him-5(e1490) V.*	This study
OH18061	*flp-7(syb5413[flp-7:sl2:GFP::H2B]) X; otIs669 him-5(e1490) V*	This study
MCP385	*twk-47(bab385[twk-47::wrmScarlet]) I*	Gift from T. Boulin lab
PHX4374	*flp-32(syb4374[flp-32:SL2:GFP::H2B]) X.*	PMID: 37935195
PHX6148	*nlp-50(syb6148[nlp-50::sl2::gfp::h2b]) II*	PMID: 34759317
PHX4512	*nlp-69(syb4512[nlp-69::sl2::gfp::h2b]) V*	PMID: 36067313
PHX7768	*tdc-1(syb7768[tdc-1::sl2::gfp::h2b]) II*	PMID: 38895397
PHX6486	*cat-1(syb6486[cat-1::SL2::gfp::H2B]) X.*	PMID: 38895397
PHX4491	*unc-17(syb4491[unc-17::T2A::GFP:H2B]) IV*	PMID: 35324425
PHX4595	*tkr-1(syb4595 [tkr-1::SL2::GFP::H2B]) III*	PMID: 37935195
OH18107	*unc-42(ot986) [unc-42::GFP] V; him-8(e1489) IV*	PMID: 34165428
OH16380	*nlp-45(ot1032[nlp-45::T2A::GFP::H2B]) X*	PMID: 34759317
OH14070	*bnc-1(ot845[bnc-1::mNeonGreen::AID]) V.*	PMID: 28056346
