# Supplementary material for: Functional analysis of conserved C. elegans bHLH family members uncovers lifespan control by a peptidergic hub neuron
Source: PLoS Biol. 2025 Jan 6;23(1):e3002979. doi: 10.1371/journal.pbio.3002979 (PMC11703107; doi:10.1371/journal.pbio.3002979)
Supplement: S2 Table — (PDF) [file pbio.3002979.s007.pdf]

Table S2: Lifespan analysis

Fig.9B (repeat1 2023.7.3)

Genotype	No. total	No. dead	No. censored	Meanlifespan (Days)	S.E.	p-value(compared w/ N2)	Bonferroni p-value(compared	Culturing temperature
N2	105	83	22	12.14	0.48			20°C
hlh-15(tm1824)	105	76	29	15.34	0.61	0.0001	0.0002	20°C
hlh-15(ot1389)	105	74	31	16.08	0.7	0.0000059	0.000012	20°C

(repeat2 2023.10.2)

Genotype	No. total	No. dead	No. censored	Meanlifespan (Days)	S.E.	p-value(compared w/ N2)	Bonferroni p-value(compared	Culturing temperature
N2	105	78	27	14.54	0.39			20°C
hlh-15(tm1824)	105	58	47	16.71	0.44	0.0001	0.0002	20°C
hlh-15(ot1389)	105	59	46	17.08	0.41	0.000011	0.000022	20°C

Fig.9G (repeat1 2024.4.2)

Genotype	No. total	No. dead	No. censored	Meanlifespan (Days)	S.E.	p-value	Bonferroni p-value	Culturing temperature
N2	105	80	25	17.56	0.48			20°C
hlh-15(ot1389)	105	44	61	19.25	0.76	0.0336(v.s. N2)	0.1343(v.s. N2)	20°C
hlh-15(ot1389) AVK::ghlh-15 #1	105	61	44	17.13	0.6	0.0269(v.s. ot1389)	0.1078(v.s. ot1389)	20°C
hlh-15(ot1389) AVK::ghlh-15 #2	105	55	50	17.38	0.59	0.046(v.s. ot1389)	0.1839(v.s. ot1389)	20°C

(repeat2 2024.5.20)

Genotype	No. total	No. dead	No. censored	Meanlifespan (Days)	S.E.	p-value	Bonferroni p-value	Culturing temperature
N2	105	77	28	16.32	0.43			20°C
hlh-15(ot1389)	105	63	42	18.55	0.45	0.0029(v.s. N2)	0.0174 (v.s. N2)	20°C
hlh-15(ot1389) AVK::ghlh-15 #1	105	68	37	16.88	0.5	0.0452(v.s. ot1389)	0.2714(v.s. ot1389)	20°C
hlh-15(ot1389) AVK::ghlh-15 #2	105	64	41	14.99	0.41	<0.0001(v.s. ot1389)	<0.0001(v.s. ot1389)	20°C

Fig.9H (repeat1 2024.5.20)

Genotype	No. total	No. dead	No. censored	Meanlifespan (Days)	S.E.	p-value	Bonferroni p-value	Culturing temperature
AVK::Cre(+) [AVK-specific egl-3 Knockout(+)]	105	74	31	16.22	0.37			20°C
Sibling(-)	105	79	26	14.61	0.34	0.0025	0.0152	20°C

(repeat2 2024.7.29)

Genotype	No. total	No. dead	No. censored	Meanlifespan (Days)	S.E.	p-value	Bonferroni p-value	Culturing temperature
AVK::Cre(+) [AVK-specific egl-3 Knockout(+)]	105	62	43	17.23	0.46			20°C
Sibling(-)	105	71	34	15.97	0.33	0.021	0.042	20°C
